# A minimum data set—Core outcome set, core data elements, and core measurement set—For degenerative cervical myelopathy research (AO Spine RECODE DCM): A consensus study

**DOI:** 10.1371/journal.pmed.1004447

**Published:** 2024-08-22

**Authors:** Benjamin M. Davies, Xiaoyu Yang, Danyal Z. Khan, Oliver D. Mowforth, Alvaro Y. Touzet, Aria Nouri, James S. Harrop, Bizhan Aarabi, Vafa Rahimi-Movaghar, Shekar N. Kurpad, James D. Guest, Lindsay Tetreault, Brian K. Kwon, Timothy F. Boerger, Ricardo Rodrigues-Pinto, Julio C. Furlan, Robert Chen, Carl M. Zipser, Armin Curt, James Milligan, Sukhivinder Kalsi-Rayn, Ellen Sarewitz, Iwan Sadler, Tammy Blizzard, Caroline Treanor, David Anderson, Nader Fallah, Olesja Hazenbiller, Carla Salzman, Zachary Zimmerman, Anne M. Wandycz, Shirley Widdop, Margaret Reeves, Rye Raine, Sukvinder K. Ryan, Ailish Malone, Ali Gharooni, Jefferson R. Wilson, Allan R. Martin, Michael G. Fehlings, Angus G. K. McNair, Mark R. N. Kotter, on behalf of the AO SPINE RECODE-DCM Steering Committee and AO Spine RECODE DCM Community

**Affiliations:** 1 Division of Neurosurgery, Department of Clinical Neurosciences, University of Cambridge, Cambridge, United Kingdom; 2 Myelopathy.org, Cambridge, United Kingdom; 3 Department of Neurosurgery, National Hospital for Neurology and Neurosurgery, London, United Kingdom; 4 Wellcome/EPSRC Centre for Interventional and Surgical Sciences, University College London, London, United Kingdom; 5 School of Medical Sciences, Faculty of Biology, Medicine and Health, The University of Manchester, Manchester, Manchester, United Kingdom; 6 Department of Neurosurgery, Geneva University Hospital, University of Geneva, Geneva, Switzerland; 7 Department of Neurological Surgery, Thomas Jefferson University, Philadelphia, Pennsylvania, United States of America; 8 Department of Neurosurgery, University of Maryland School of Medicine, Baltimore, Maryland, United States of America; 9 Department of Neurosurgery, Sina Trauma and Surgery Research Centre, Tehran University of Medical Sciences, Tehran, Iran; 10 Department of Neurosurgery, Medical College of Wisconsin, Wauwatosa, Wisconsin, United States; 11 Department of Neurosurgery and The Miami Project to Cure Paralysis, The Miller School of Medicine, University of Miami, Miami, Florida, United States; 12 Department of Neurology, New York University Langone Health, New York, New York, United States of America; 13 Department of Orthopaedics, International Collaboration on Repair Discoveries (ICORD), Faculty of Medicine, University of British Columbia, Vancouver, Canada; 14 Spinal Unit (UVM), Department of Orthopaedics, Centro Hospitalar Universitário do Porto - Hospital de Santo António, Porto, Portugal; 15 Instituto de Ciências Biomédicas Abel Salazar, Porto, Portugal; 16 Department of Medicine, Division of Physical Medicine and Rehabilitation, University of Toronto, and KITE Research Institute and Toronto Rehabilitation Institute, University Health Network, Toronto, Canada; 17 Division of Neurology, University of Toronto, Toronto, Canada; 18 University Spine Centre, Balgrist University Hospital, Zurich, Switzerland; 19 Department of Family Medicine, McMaster University, Hamilton, Canada; 20 KITE Research Institute, University Health Network, Toronto, Canada; 21 School of Health Sciences, Faculth of Medicine and Health, University of Sydney, Sydney, Australia; 22 Praxis Spinal Cord Institute, Vancouver, Canada; 23 AO spine, Davos, Switzerland; 24 Royal College of Surgeons in Ireland, Dublin, Ireland; 25 Division of Neurosurgery, Department of Surgery, University of Toronto, Toronto, Canada; 26 Department of Neurological Surgery, University of California, Davis, Sacramento, California, United States of America; 27 Centre for Surgical Research, Bristol Medical School: Population Health Sciences, University of Bristol, Bristol, United Kingdom

## Abstract

**Background:**

Degenerative cervical myelopathy (DCM) is a progressive chronic spinal cord injury estimated to affect 1 in 50 adults. Without standardised guidance, clinical research studies have selected outcomes at their discretion, often underrepresenting the disease and limiting comparability between studies. Utilising a standard minimum data set formed via multi-stakeholder consensus can address these issues. This combines processes to define a core outcome set (COS)—a list of key outcomes—and core data elements (CDEs), a list of key sampling characteristics required to interpret the outcomes. Further “how” these outcomes should be measured and/or reported is then defined in a core measurement set (CMS). This can include a recommendation of a standardised time point at which outcome data should be reported. This study defines a COS, CDE, and CMS for DCM research.

**Methods and findings:**

A minimum data set was developed using a series of modified Delphi processes. Phase 1 involved the setup of an international DCM stakeholder group. Phase 2 involved the development of a longlist of outcomes, data elements, and formation into domains. Phase 3 prioritised the outcomes and CDEs using a two-stage Delphi process. Phase 4 determined the final DCM minimal data set using a consensus meeting. Using the COS, Phase 5 finalised definitions of the measurement construct for each outcome. In Phase 6, a systematic review of the literature was performed, to scope and define the psychometric properties of measurement tools. Phase 7 used a modified Delphi process to inform the short-listing of candidate measurement tools. The final measurement set was then formed through a consensus meeting (Phase 8). To support implementation, the data set was then integrated into template clinical research forms (CRFs) for use in future clinical trials (Phase 9).

In total, 28 outcomes and 6 domains (Pain, Neurological Function, Life Impact, Radiology, Economic Impact, and Adverse Events) were entered into the final COS. Thirty two outcomes and 4 domains (Individual, Disease, Investigation, and Intervention) were entered into the final CDE. Finally, 4 outcome instruments (mJOA, NDI, SF-36v2, and SAVES2) were identified for the CMS, with a recommendation for trials evaluating outcomes after surgery, to include baseline measurement and at 6 months from surgery.

**Conclusions:**

The AO Spine RECODE-DCM has produced a minimum data set for use in DCM clinical trials today. These are available at https://myelopathy.org/minimum-dataset/. While it is anticipated the CDE and COS have strong and durable relevance, it is acknowledged that new measurement tools, alongside an increasing transition to study patients not undergoing surgery, may necessitate updates and adaptation, particularly with respect to the CMS.

## Introduction

Degenerative cervical myelopathy (DCM) is a progressive subacute to chronic spinal cord injury caused by narrowing of the cervical canal with static and dynamic cord compression [1,2]. Symptoms can include loss of dexterity, imbalance, falls, and/or pain [1]. Although DCM is estimated to affect as many as 1 in 50 adults [3], less than 1 in 10 are diagnosed today [4]. Treatment is currently limited to surgery, and while meaningful, recovery is most often incomplete [5]; dependence and unemployment are high, and patients face life-long disability [6]. These effects translate into some of the poorest quality of life scores of any chronic disease [7]. Research that can improve outcomes is urgently required.

Although progress is being made in improving patients’ quality of life [8], it is apparent further success in DCM research is hindered by many inefficiencies, including inconsistent data reporting between studies [9], creating challenges for aggregate analysis, but also commonly overlooking patient priorities [10]. The impact of these inefficiencies is compounded by the relative size of the research field [11]. In a context, based on a comparative search of DCM, multiple sclerosis, amyotrophic lateral sclerosis, and spinal cord injury using a platform called Dimensions.ai demonstrates that since 2011, DCM has received less than 2% of grant funding awarded compared to these other diseases [11].

AO Spine RECODE-DCM (Research Elements and Common Data Elements) was established to tackle some of these inefficiencies [12], with the overall aim of accelerating knowledge discovery to improve outcomes. This initiative included establishing a unifying term [2] and definition for the disease, establishing the top 10 research priorities [6,11,13–23], and defining a minimum data set for research. Minimum data sets are consensus agreed datapoints that should be reported as a minimum in all clinical trials. Supported by methodological research groups such as the Core Outcome Measures in Effectiveness Trials (COMET, comet-initiative.org), they have a well-established approach to address inconsistent data reporting across a field. This process includes forming a core outcome set (or comment outcome set, COS)—a list of critical outcomes that should be measured, and the core data elements (or comment data elements, CDE)—a list of the critical sampling characteristics that should be reported to enable interpretation of outcomes. While this defines “what” to report, for consistency, it is also important to establish “how” outcomes should be measured. This final step is called a core measurement set (CMS) [24].

This article outlines the multi-stakeholder consensus process to define a minimum data set for clinical research in DCM. It presents both “what” (COS and CDE) to measure, as well as “how” (CMS). This was an intensive and iterative process. The key recommendations, including template clinical research forms, are also consolidated at https://myelopathy.org/minimum-dataset/.

## Methods

This individual project falls under the greater AO Spine RECODE DCM (aospine.org/recode) initiative, for which the protocols [25,26] and other elements have been published [2,12,27,28].

Briefly, the minimum data set was defined using a modified Delphi process, which iteratively examines consensus from a relevant group [29], Phase 1 involved the setup of an international DCM stakeholder group. Phase 2 involved the development of a longlist of outcomes and data elements and formation into domains. Phase 3 prioritised the outcomes and core data elements using a two-stage Delphi process. Phase 4 determined the final DCM minimal data set using a consensus meeting. Using the COS, Phase 5 created definitions of the measurement construct for each outcome. Phase 6 undertook a systematic review of the literature, to define the psychometric properties of currently used measurement tools. Phase 7 used a modified Delphi process to inform the short-listing of candidate measurement tools. The final measurement set was formed using a consensus meeting (Phase 8). This included recommendations on the timing of assessments, which was supported by an expert review of recovery trajectories after surgery based on predefined and existing data sets. To support implementation, the CMS and CDE were then integrated into template clinical research forms (CRFs) for use in future clinical trials (Phase 9).

This methodology was developed with guidance from COMET [30], Outcomes Measurement In Rheumatology (OMERACT) (omeract.org), and the Consensus-based Standards for the selection of health Measurement Instruments (COSMIN) (cosmin.nl). The reporting here therefore aligns with the Core Outcome Set-STAndards for Reporting (COS-STAR) framework (S1 Data) [31].

### Phase 1—Stakeholders, oversight, and approvals

This project has defined 3 key stakeholder groups: spinal surgeons, other healthcare professionals (oHCP), and patients with DCM (PwCM) [32] or their care providers. The project was overseen by an international steering committee (SC) (S2 Data). The project had oversight provided by a management group comprised of 1 spinal surgeon (MRK), 3 surgeons-in-training (BMD, DZK, and ODM), 2 individuals with DCM (IS and ES) and a project manager (OH). Ethical approval for the study was granted by the University of Cambridge (HBREC.2019.14).

### Phase 2—Generation of longlist of outcomes, data elements, and formation into domains

The longlist was developed using several approaches.

#### 2.1 Systematic reviews of the literature

Systematic reviews of EMBASE, MEDLINE, and the Cochrane Register of Clinical Trials were performed to identify outcome measures and domains, and data elements used within primary DCM clinical studies published between 1995 and 2015. Content was further analysed to identify key themes of the identified outcomes and data elements. These reviews were supplemented by a previously published systematic review that focused on surgical complications [33]. EMBASE, MEDLINE, and the Cochrane Register of Clinical Trials were queried from their inception to June 2016. Details of these reviews have been previously published [9,33,34].

#### 2.2 Lived experience perspective: Content analysis of interviews and a survey

To capture the experience of PwCM and their supporters, a focus group was held which underwent content and thematic analysis and was used to develop a survey for PwCM to gain further insights. The members of the focus group participated in a semi-structed interview conducted by BMD and MRK. The lists of generated outcomes and domains were further reviewed with removal of duplicates. A survey was developed with SurveyMonkey (California, United States of America) to explore these outcomes in a larger sample of PwCM and disseminated using Myelopathy.org charity via email and social media platforms to increase uptake. Further details of the survey methodology have previously been published [13]. Recordings of the focus group also underwent thematic analysis using NVivo software (version 10, 2012, QSR International Pty, Victoria, Australia) to identify any implicit outcome measures that may have otherwise not been captured in content analysis or the survey. Further details on this analysis and methodology have been previously published [35]. The outcomes and data elements identified from the systematic reviews, focus groups, patient survey, and thematic analysis were reviewed and refined by the stakeholder SC to generate a longlist of outcomes and data elements.

#### 2.3 Finalising an initial longlist

The results from these outcomes and domains-generating processes were reviewed and refined by the Management Group (S3 Data), with the aim of generating a longlist of unique outcomes and domains of relevance to a DCM COS. This was an iterative process, conducted using virtual teleconference and a shared spreadsheet, until all members are satisfied. The eventual list was placed into the Round 1 survey.

### Phase 3—Interim prioritisation

#### 3.1 Delphi survey, Round 1

The longlist of outcomes generated in Phase 2 were put forward for prioritisation using a two-round Delphi survey to achieve consensus on core outcomes and data elements. The survey was handled using Surveylet (Calibrum, San Francisco, USA). The survey was distributed to key stakeholders internationally including spinal surgeons, oHCP, and PwCM/supporters. The survey was divided into 2 parts: the candidate outcomes and the candidate data elements. PwCM/supporters completed only the outcomes component, as it was decided that only spinal surgeons and the oHCP had the correct perspective for selecting sampling characteristics. The dissemination strategy is published elsewhere [13]. Survey participants used the GRADE (Grading of Recommendations, Assessment, Development and Evaluations) approach [36] and were asked to score outcomes for inclusion into COS on a scale of 1 to 9, with 1 being not important at all and 9 being the most important. The survey also allowed for additional suggestions of outcomes by participants. Spinal surgeons and oHCP were also asked to grade data elements identified in Phase 2 as part of the same survey.

Following closure of the survey, additional outcomes were reviewed by the multi-stakeholder AO Spine RECODE-DCM Management Group, to identify in-scope and otherwise unrepresented outcomes. Any new suggestions were then reviewed at an AO Spine RECODE-DCM SC meeting to approve those that would enter the Round 2 survey.

#### 3.2 Delphi survey, Round 2

The same stakeholders were invited to complete Round 2 of the Delphi survey. Feedback from Round 1 was provided, including overall scores, scores based on stakeholder for outcomes, and ratings of outcomes. New outcomes were presented as per Round 1. Participants again were required to rate outcomes using the GRADE system. The same framework and survey were used for data elements; however, only clinicians and researchers were asked to complete this part.

Each outcome present in Round 1 included a bar chart, displaying the aggregate results per stakeholder group for “Balance.” In addition, the outcome rating was pre-populated with the participants’ previous rating for reference. As new outcomes lacked any preexisting data, these questions were unfilled and displayed without a graph.

A priori consensus criteria were established at the start of the project. Specifically, for “consensus in,” an outcome or data element required a stakeholder group to either score ≥70% 7–9 and ≤15% 1–3, with ≥50% score 7–9 per remaining stakeholder groups or a stakeholder group to score ≥90% 7–9. For “consensus out,” a threshold of ≤15% score 7–9 and ≥70% score 1–3 in a single stakeholder group, with ≥50% score 1–3 per remaining stakeholder groups.

### Phase 4—Final prioritisation: COS and CDE

A final consensus meeting was held virtually over Zoom (California, USA) in October 2020 to finalise the COS and CDE. This was a deviation from the original “in person” meeting plans, due to the COVID-19 pandemic. Participants included spinal surgeons, oHCP, and PwCM/supporters. The meeting was facilitated by 3 trained facilitators and comprised of the following format:

#### Pre-consensus meeting survey

Participants attending the consensus meeting were asked to grade each of the outcomes that did not achieve consensus (“no consensus”) from Phase 3 as “core,” “not core,” or “unsure” prior to the meeting. Outcomes were presented under corresponding domains, and data from second round Delphi survey was presented (i.e., grade ratings per stakeholder group). The aim of this exercise was to familiarise participants with outcomes under “consensus in” and “no consensus” and generate initial steer prior to the meeting. The results were not used to define consensus in or out.

#### Consensus meeting

The final consensus meeting incorporated the use of “breakout rooms,” where 3 smaller groups (equal balance of stakeholder groups) were formed to enable greater discussion and contribution, in 3 sessions each facilitated by one of the trained facilitators. In each group’s session, an interactive screen distinguishing areas for “consensus out,” “undecided,” or “consensus in” was provided with the facilitator moving undecided outcomes based on group discussions and majority decision. The 3 groups reconvened for further discussion on outcomes, with “consensus in” defined as the outcome reaching “core” for each focus group (i.e., all 3 groups agree for inclusion “core”).

Following the COS consensus meeting, spinal surgeons and oHCP in the meeting participated in a further session to discuss data elements. Similar to the COS, data elements had been rated prior to the meeting, with results available for review. Discussion and voting for inclusion or exclusion of data elements was facilitated by trained facilitators to develop final CDE.

### Phase 5—Define measurement constructs and preferred measurement approach

Draft definitions were generated from original source documents including published literature or interviews with patients and professionals. These provisional definitions were then reviewed by the SC and iterated as indicated. Furthermore, for each outcome domain, the SC were asked to provide a steer as to whether it should be measured by people with DCM (i.e., a patient reported outcome measure or PROM), a healthcare professional (i.e., a clinician reported outcome measure or ClinROM), or both.

### Phase 6—Identifying potential instruments and their measurement properties

A search was performed in EMBASE and MEDLINE from inception until 4 August 2020 to identify original research assessing the measurement properties of instruments used in clinical research of DCM [28]. The search string was built using the relevant DCM search filter [37] and the COSMIN filter for studies evaluating measurement properties [38]. Abstracts were screened by 4 reviewers against a set of predefined criteria (Table 1). Only primary clinical research studies evaluating one or more measurement properties were included.

**Table 1:** Inclusion and exclusion criteria for CMS systematic review.

Inclusion	Exclusion
Publication type	
Article written in English Primary clinical research articles	Article not written in English Conference abstracts or posters Editorials, commentaries, opinion papers, or letters Book chapters or theses
Study type	
Study includes primary clinical data	Study uses only secondary data Case reports Narrative reviews Systematic reviews Meta-analyses
Populations	
Human studies	Nonhuman studies
Indications	
Exclusively DCM (CSM, OPLL, cervical stenosis, spondylosis, spinal cord compression, cervical myelopathy)	Populations with DCM and at least one other condition (e.g., radiculopathy)
Comparator	
At least 1 assessment tool from [9,39,40]	
Outcomes	
At least 1 psychometric property At least 1 MCID or SCB	

CMS, core measurement set; CSM, cervical spondylotic myelopathy; DCM, degenerative cervical myelopathy; MCID, minimally clinical important difference; OPLL, ossification of the posterior longitudinal ligament; SCB, substantial clinical benefits.

All data were collected, processed, and analysed in accordance with the COSMIN manual for systematic reviews of PROMs. This included a quality assessment using the COSMIN risk of bias checklist [41–43] and collecting results across 10 measurement properties: content validity, structural validity, internal consistency, cross-cultural validity/measurement invariance, reliability, measurement error, criterion validity, hypotheses testing for construct validity, responsiveness, and clinically important differences. Results were rated as “sufficient,” “indeterminate,” or “insufficient” and overall methodological quality scores were scored as “very good,” “adequate,” “doubtful,” “inadequate,” or “not applicable.” Results were then qualitatively summarised and an overall rating of the quality of the studies was made using a modified GRADE approach.

### Phase 7—Shortlisting candidate measures

#### 7.1 Gap analysis

Instruments meeting the recommendation threshold were matched against the COS by construct. To identify candidate instruments for outcomes without a listed instrument, searches were conducted outside of DCM. Initially, this was looked at pragmatically using MEDLINE, to establish if any such review already existed [25].

#### 7.2 Scoping reviews of related neurological disease

**Fig 1:**
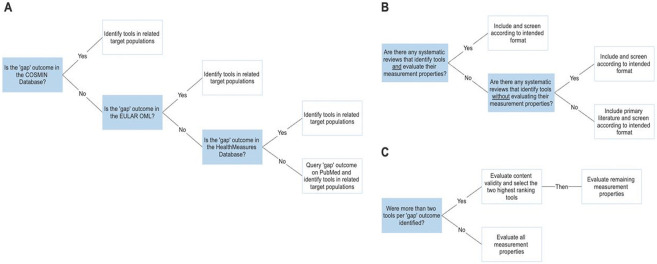
Decision algorithm to select candidate instruments from outside of DCM: (A and B) Stage 1: selection of databases for identification of tools outside DCM (A) and screening of tools outside DCM (B). (C) Stage 2: evaluation of measurement properties. COSMIN, Consensus-based Standards for the selection of health Measurement Instruments; DCM, degenerative cervical myelopathy; OML, Outcomes Measures Library.

For those remaining outcomes without potential instruments, focused scoping reviews were conducted to identify instruments used in a related target population and to evaluate their quality. Given the intensive undertaking of reviewing the quality of instruments using the COSMIN methodology, a pragmatic approach was developed to ensure this undertaking was manageable and likely to yield relevant results (Fig 1):

Step 1: Scoping
  1a Identify candidate tools from outside DCM, for each gap outcome.
  1b Include tools based on “who” they should be performed by (ClinROM versus PROM) defined during Stage 1 by the SC.
Step 2: Shortlisting
  2a Evaluate content validity.
  2b Shortlist up to 2 instruments per gap outcome.
  2c Evaluate measurement properties of instrument as per COSMIN.

Specifically, each “gap” outcome was first queried in the COSMIN database of systematic reviews of outcome measurement instruments (https://database.cosmin.nl/) (Fig 1A). The above steps were performed for each “gap” outcome. If no candidate instruments were found through the COSMIN database, the same steps were performed on the EULAR Outcomes Measures Library (OML, https://oml.eular.org/) (Fig 1A). If no such instruments were found through the EULAR OML, the same search was performed on the HealthMeasures Database (https://www.healthmeasures.net/) or MEDLINE using the COSMIN filter. These databases were selected based on their scope. Suggestions from the SC were also considered.

Only instruments whose category matched the intended measurement method (e.g., PROM by the patient versus ClinROM by the professional) as defined in Stage 1 were included. For example, if “faecal incontinence” was defined as a patient-reported outcome during Phase 1, then only PROMs of “faecal incontinence” were included, and ClinROMs were excluded. Reviews must target disease populations related to DCM to increase the likelihood of content validity. For example, “faecal incontinence,” could be a symptom of many diseases. However, since this symptom is also measured in other spinal disorders with neurological injury (e.g., traumatic spinal cord injury and cauda-equina syndrome), these disorders would be considered appropriate populations.

The methodological quality of identified instruments was evaluated using the standard COSMIN process. Recognising that evaluating an uncapped number of instruments with the COSMIN manual can be unrealistic, the number of instruments for COSMIN review was limited to 2 per “gap” outcome. If there were more than 2 PROMs or ClinROMs per “gap” outcome, a content validity survey was conducted with at least 5 stakeholders to rank the identified instruments (Fig 1C). The 2 highest ranking instruments underwent COSMIN evaluation.

### Phase 8—Consensus

#### 8.1 Formation of an expert consensus panel

A multidisciplinary panel of experts was formed to finalise the CMS through consensus. These experts were identified using purposive sampling to include people with lived experience; professionals from key clinical disciplines commonly involved in DCM care (i.e., spinal surgery, neurology, rehabilitation medicine, physiotherapy, and primary care); professionals with clinical trials experience, particularly with respect to measuring each of the 6 domains (i.e., adverse events, economic impact, life impact, neurological function, pain, and radiology); and professionals with experience in trial statistics. It was agreed that at least half of all participants would be external to the SC; at least 1 in 5 participants would have lived experience; and no more than half of all participants would be spinal surgeons. It also aimed to have a 1:1 ratio of women to men. All panellists must have declared any conflicts of interest, and been approved by the SC.

#### 8.2 Pre-meeting short-listing

Panellists were provided with a summary of the measurement instruments considered of sufficient quality for each element, along with their evidence base, and the original SC decision concerning the preferred reporting method (i.e., PROM or ClinROM). Panellists reviewed this in advance of the meeting and were asked to submit up to 2 instruments shortlisted, or 2 instruments from their experience, per domain. To justify the suggestion of instruments from outside the provided list, panellists were asked to cite 1 primary article per psychometric domain (i.e., 1 for validity, 1 for reliability, and 1 for responsiveness). Any new suggested tools underwent COSMIN evaluation.

#### 8.3 Face-to-face consensus meeting

A consensus meeting was then convened. Each domain was discussed with >70% agreement considered consensus. The consensus meeting was overseen by an independent facilitator and followed a nominal group technique. Moderated discussion and re-voting were undertaken as necessary until consensus was achieved for all components of the COS and CDE.

Following the selection of the final instruments, a presentation was given by an independent expert (AM) to summarise the known recovery profiles following surgery for DCM. Data was principally drawn from AO Spine North America and World prospective observational studies, and the Canadian Spine Outcomes and Research Network (CSORN, www.csorncss.ca). These data sets reported on longitudinal recovery after surgical intervention and were pragmatically selected given their high-quality and proven generalisation. The objective was to recommend one time point at which outcomes should always be reported in DCM trials evaluating recovery after surgery, recognising that the amount of recovery varies with time from surgical intervention, and could be another source of variability when comparing studies.

### Phase 9—Implementation

A set of CRFs were created according to the developed COS and CDE using Microsoft Publisher (version 2019, Microsoft Corporation, Washington, USA), based in those in use for ongoing DCM trials; RECEDE-Myelopathy [44] and POLYFIX-DCM (S5 Data).

## Results

### Core outcome set

#### Generation of longlist

A systematic review (*n* = 108) was performed to identify the outcomes measures [9]. A further review (*n* = 42) was conducted to analyse the complications of DCM surgery [33].

Furthermore, a focus group of PwCM/supporters participated in semi-structured discussion: 8 individuals were involved, 5 had DCM (3 males, 2 females), and 3 were friends/family (all women). The workshops generated 52 unique outcomes [45]. Interestingly, supporters had separately identified 4 problems not reported by PwCM: difficulty initiating urination, loss of coordination, inability to make plans, and altered cognition.

A survey was then developed to explore these outcomes in a large sample. The 52 outcomes were split into “symptoms” and “life impact,” to add some structure. The survey was accessed 294 times, including 8 duplicate entries and 62 incomplete entries. Therefore, 224 responses underwent analysis. The respondents were on average 56.6 years old, had lived with DCM for 8.2 years, and had a modified Japanese Orthopaedic Association (mJOA) score of 11.6. Respondents were more likely to be female (76%) and to have undergone surgery (62%). All outcomes were experienced by at least 5% of participants surveyed [45]. Erectile dysfunction was reported only by male respondents (34% of male respondents), confirming internal consistency. Gender, surgical history, disease severity (mJOA), or time lived with DCM did not influence the distribution of outcomes reported.

Nearly half (*n* = 94, 42%) of respondents suggested additional outcomes, the majority of which were felt to already be included. Those omitted were added to the longlist of outcomes.

The above results were reviewed and refined by the Management Group and a list of 63 outcomes was generated and placed into the Round 1 survey.

The outcomes were aggregated thematically by the study authors, with reference to the OMERACT filter [46]: Pain, Neurological Function, Quality of Life, Imaging, and Surgical Complications. These were used to structure the survey.

#### Interim prioritisation

In Round 1, 332 stakeholders participated in the COS survey: 113 (34%) PwCM or their supporters, 158 spinal surgeons (48%), and 61 (18%) oHCP. Detailed sampling demographics are found in S4 Data.

oHCP suggested 50 additional outcomes, while PwCM or their supporters recommended 64. Follow processing and committee review, 9 additional outcomes were identified that were not otherwise included in Round 1: Chest Pain, Tremor, Shoulder Mobility, Neck Mobility, Visual symptoms, Cognition/Confusion, Stamina, Length of Hospitalisation, and Horner’s Syndrome (a surgical complication).

85% spinal surgeons, 84% PwCM and their supporters, and 82% oHCP completed the second round of the Delphi survey. A total of 28 outcomes had reached the “consensus in” threshold, and 0 outcomes the “consensus out” threshold, leaving 44 outcomes for further review.

The original aim for the COS was to contain 15 outcomes, which had been proposed based on the experience of other COS in order to reduce the likelihood that the COS became onerous to incorporate in trials. Furthermore, considering 44 outcomes using a virtual consensus meeting would be challenging. Consequently, the SC considered areas for additional interim guidance that could either consolidate outcomes and/or provide valid consensus decisions on outstanding outcomes: the surgical complications were reviewed. In this category, 9 out of 13 outcomes had achieved “consensus in,” with the remainder achieving >50% Grades 7 to 9 rating for 2 out of the 3 stakeholder groups. Each of these outcomes was very specific, and it was felt this granularity could be better incorporated into the definition or measurement approach. Consequently, surgical complications were merged to form 1 core outcome. Death was considered a separate core outcome. Further refinement was not considered possible at this stage, and all the other outstanding outcomes were discussed during a formal consensus meeting as planned.

#### Final prioritisation

Overall, the consensus meeting included 24 participants (13 [54%] from the AO Spine RECODE-DCM Steering Committee): 11 PwCM, 7 spinal surgeons, and 6 oHCP.

In total, 28 outcomes and 6 domains (Pain, Neurological Function, Life Impact, Radiology, Economic Impact, and Adverse Events) were entered into the final COS. Within the Life Impact domain, the groups identified that many of the outcomes triangulated into 2 broader groups: Fatigue, capturing vitality, stamina, retribution, and variability; and Mental Health, capturing frustrations, relationship difficulties, anticipatory anxiety, and helplessness. Within Neurological Function, neck mobility was considered a “core” outcome. Likewise, paraesthesia was identified as a “core” outcome; however, the group agreed that this symptom existed on a spectrum with numbness and would be better assessed as sensory dysfunction. Urinary outcomes were also regrouped under a single outcome, bladder dysfunction. Within Economic Impact, both cost of care and employment status were considered “core.” Within Radiology, cervical spine alignment was considered “core” but required further discussion and a vote during the plenary session. No further outcomes were included within Adverse Events.

**Fig 2:**
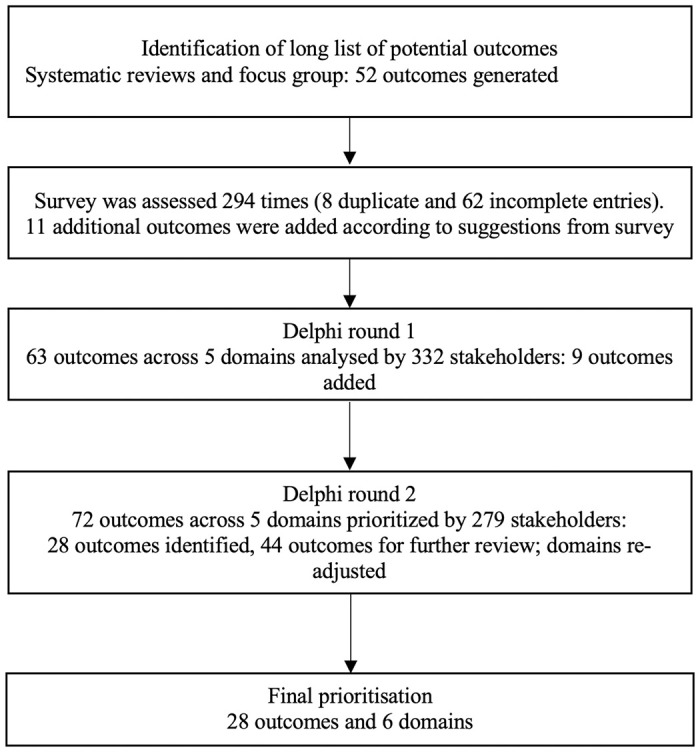
Summary of the development of a COS. COS, core outcome set.

Selecting outcomes for the Pain domain generated significant discussion. While the original process had focused on pain location over perception, the group agreed that this was, in fact, counter-intuitive and strongly informed by how pain was measured in the past (e.g., Neck and Arm Pain). The group agreed that pain outcomes should include location, perception, and intensity, along with the already “consensus in” Pain Control. Further, muscle spasms/shaking were considered a manifestation of spasticity and therefore moved from the Pain domain to the Neurological Function domain and considered more broadly as muscle tone. This was supported by PwCM who confirmed these involuntary movements were more an inconvenience than a pain. The final COS is shown in Table 2. The summary of the development of a COS is shown in Fig 2.

**Table 2:** Core outcome set: The set includes 6 domains and 28 outcomes.

Domain	Outcome	Lay description
**Adverse events**	Death	Defined as the point at which all the body’s organs cease working permanently.
	Surgical adverse events	Any event that is due to medical or surgical management and not due to the underlying disease process or injury, which leads to harm of the patient or requires additional monitoring or treatment.
**Economic impact**	Employment status	Current occupation (paid or unpaid, job-seeking and/or welfare support status).
	Cost of care	The total costs attributable to the disease from a societal perspective (to therefore include both the cost of providing a treatment or care [Formal Health Sector Perspective], with the associated costs experienced by the patient and/or their household, such as informal care, loss of productivity, and change in employment [informal Health Sector Perspective and Non-Health Sector Costs]).
**Life impact**	Falls	The occurrence of fall(s); events at which a person comes to rest inadvertently on the ground or floor or other lower level.
	Mobility	The ability to move in one’s environment with ease and without restrictions safely, regularly, repeatedly, and in a timely manner.
	Dependence	The level of independence, i.e., the need and/or reliance on another’s for physical, financial, or emotional support, to manage activities of day-to-day life.
	Fatigue	A feeling, often articulated as tiredness, a lack of energy or exhaustion, that manifests as difficulty with physical or cognitive activity.
	Mental health	The psychological, emotional, and social well-being of an individual.
**Neurological function**	Sensory dysfunction	Loss of normal sensation to superficial surfaces (e.g., skin or mucous membranes), including an absence or reduction (e.g., numbness) to touch, and perception of altered sensations without stimulation (e.g., paraesthesia).
	Bladder Dysfunction	Loss of normal bladder function, which can include difficulty passing urine (e.g., initiating urination, altered flow and incomplete voiding), nocturia (e.g., increased requirement to urinate overnight), and incontinence (e.g., stress or urge incontinence).
	Faecal incontinence	The involuntary loss of stool that is a social or hygiene problem.
	Arm strength	Muscle strength in the arm(s) and its impact on arm functions or activities.
	Leg strength	Muscle strength in one or both legs, and its impact on leg functions or activities, such as getting up to go, standing, and walking.
	Balance	The ability to sit, stand, or walk without falling or feeling unsteady.
	Dexterity	The ability to perform small or precision tasks with the fingers and/or hands (e.g., buttoning a shirt or using a pen) safely, repeatedly, to an acceptable standard and in a timely manner.
	Finger strength	The ability of a finger to adequately apply pressure individually, or in combination with another finger, to clasp an object, regularly and repeatedly to safely perform the desired function in a timely manner.
	Grip strength	The ability for the hand, defined as the fingers in combination, to clasp or hold an object securely regularly, repeatedly, and safely.
	Neck mobility	The ability for the neck to move freely in all directions.
	Spasticity	Disordered sensorimotor control of the musculature, that can manifest as intermittent, repetitive, sustained, or unbalanced involuntary activation of muscle(s).
**Pain**	Location	Location or distribution of pain considering at least the neck, torso, arm, hand, and leg.
	Perception	The pain affect defined as the distress or unpleasant characteristics of the pain.
	Intensity	The overall severity or amount of pain.
	Pain control	The use and effectiveness of treatments to manage pain.
**Radiology**	Adjacent segment degeneration	The occurrence of new degenerative changes at a spinal level adjacent to a surgically treated level.
	Cord compression	Radiological evidence of distortion or disruption to the normal shape, contour, or position of the spinal cord.
	Cord signal change	Evidence of change within the spinal cord on imaging.
	Cervical spine alignment	The curvature of the cervical spine.

PwCM, patients with DCM.

### Core date element

#### Generation of longlist

A systematic review (*n* = 108) was performed to identify the data elements reported [34]. Reported characteristics were grouped into 3 themes: (1) study design and patient selection; (2) patient characteristics; and (3) treatment intervention(s) and course. This generated a longlist of 33 data elements, ranging from age and biological sex to co-morbidities, image findings, and surgical or nonsurgical treatment details.

#### Interim prioritisation

Following Round 1, 67 further comments were made suggesting additional data elements, of which 29 were felt to be new and unique. These were inserted into the second round Delphi survey. Following the second-round survey, 33 data elements were identified as core; specifically, 27 via consensus, and a further 6 based on literature referencing.

#### Final prioritisation

Following the virtual consensus meeting, the oHCP participated in an additional plenary a plenary session covering the remaining data elements without consensus.

**Fig 3:**
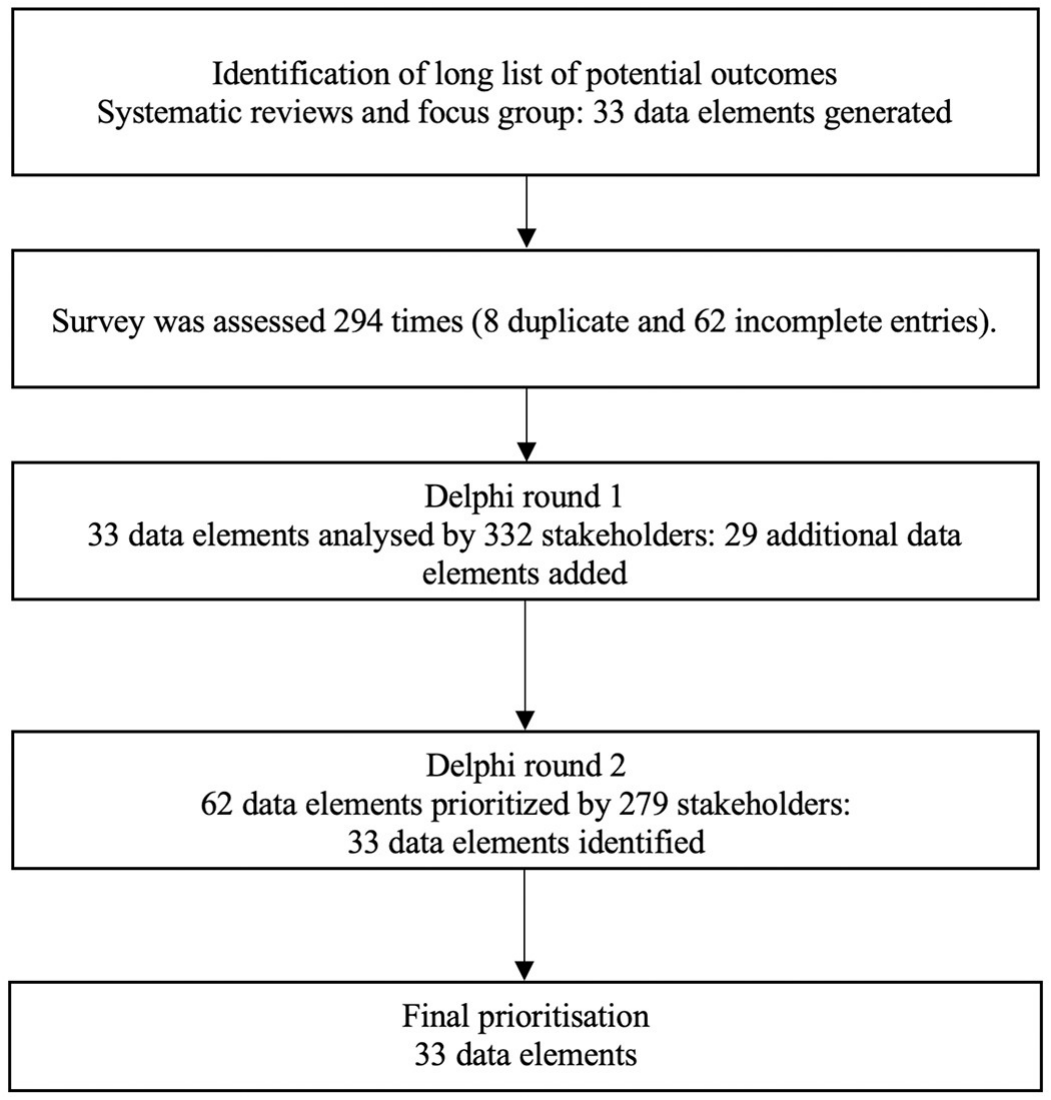
Summary of the development of a CDE. CDE, core data elements (or comment data element).

In total, an additional 6 elements were added to the CDE. In a similar fashion to the COS, grouping categories were used for the purpose of structure. In addition, as a final processing measure, any data elements which were also represented in the outcomes set were removed (on the basis that outcomes would be measured at baseline also). Thus “Deformity” was removed from the CDE, given that “Cervical Spine Alignment” had become a Core Outcome. This was performed by the Management Group and agreed by the AO Spine RECODE-DCM Steering Committee. The final CDE is shown in Table 3. The summary of the development of a CDE is shown in Fig 3.

**Table 3:** Core data element: Arranged by domain and subdomain. Each data element includes its description and the method by which its entry into the CDE was made.

Domain	Sub domain	Data element	Description	Consensus method
Individual	Demographics	Biological gender	The biological state of being male or female.	Delphi survey
		Ethnicity	Belonging to a group of people who share similar national, racial, or cultural origins.	Consensus meeting
		Age	How many years old someone is.	Literature
	Comorbidities	Mental health	The condition of emotional, psychological, and social well-being and whether or not there is any mental illness.	Literature
		Obesity	The condition defined by the accumulation and storage of excess body fat.	Literature
		Diabetes	Conditions where blood sugar levels are not controlled, because insulin either is not produced or is not used efficiently by the body.	Literature
		Smoking status	Describes whether a person inhales the fumes of burning tobacco and, if so, how much.	Literature
		Performance status	A measure of general well-being and ability to carry out the activities of daily life.	Literature
		Other neurological disease	The presence of an additional disease involving the central or peripheral nervous system and resulting in deficits in arousal, cognition, language, or motor, sensory or autonomic function of the face, arm, or legs.	Consensus meeting
Disease		Definition of DCM	Definition and/or criteria used to make a diagnosis of DCM.	Delphi survey
		Time of first symptoms	Time at which symptoms started.	Delphi survey
		Time of diagnosis	Time at which a formal diagnosis of DCM was made.	Delphi survey
		Rate of progression	The speed at which features of DCM become worse as experienced by an individual.	Delphi survey
		Number of previous surgeries	The amount of previous surgeries attempting to treat DCM.	Delphi survey
		Coexistent radiculopathy	The presence of nerve root compression in addition to spinal cord compression.	Delphi survey
Investigation	Imaging	Use of MRI imaging	The use of conventional MRI cervical spine imaging as part of diagnosis and work-up.	Delphi survey
	Imaging	Use of CT imaging	The use of CT cervical spine imaging as part of diagnosis and work-up.	Delphi survey
	Imaging	Level(s) of compression	The area(s) of compression of the spinal cord in DCM.	Delphi survey
	Imaging	Pathology causing compression	The predominant disease process causing excessive pressure on the spinal cord.	Delphi survey
	Imaging	Amount of cord compression	The extent of compression of the spinal cord in DCM.	Delphi survey
	Imaging	Presence of cord signal change	Damage and swelling of the spinal cord can appear as a change in signal on MRI scans.	Delphi survey
	Imaging	Syrinx	The presence or absence of a spinal cord syrinx.	Delphi survey
	Imaging	Spondylolisthesis	The presence or absence of spondylolisthesis.	Delphi survey
	Imaging	Radiological stability	The presence or absence of motion, as defined using dynamic imaging.	Delphi survey
	Examination	Long tract signs	Evidence from examination of spinal cord dysfunction, e.g., hyperreflexia, increased tone, or Hoffman’s sign.	Delphi survey
Intervention	Surgical	Time of treatment	The time between onset of symptoms and initiation of treatment.	Delphi survey
	Surgical	Operation type	The type of surgery used to treat DCM.	Delphi survey
	Surgical	Approach (anterior/posterior/combined)	This refers to the direction of approaching the spinal cord during surgery.	Consensus meeting
	Surgical	Operated level(s)	The area(s) of compression that hope to be relieved by surgery.	Delphi survey
	Surgical	Instrumentation	The implantation of metalwork during surgery (screws, cages, plates, etc.) to provide stability and promote bone fusion.	Delphi survey
	Surgical	Primary surgeon experience	Experience of primary surgeon.	Delphi survey
	Surgical	Postoperative rehabilitation/physiotherapy	Use of physiotherapy after an operation to help improve or restore movement and physical function.	Delphi survey

CDE, core data elements (or comment data element); DCM, degenerative cervical myelopathy.

### Core measurement set

#### Stage 1

Two SCs were required to finalise the outcome definitions and consolidate recommendations on who should measure them. At a domain level, it was felt that Adverse Events and Radiology should be evaluated using ClinROMs; Neuro-Muscular Function, Life Impact and Pain should be assessed using PROMs; and Economic Impact should require both professional and patient input.

#### Stage 2

**Fig 4:**
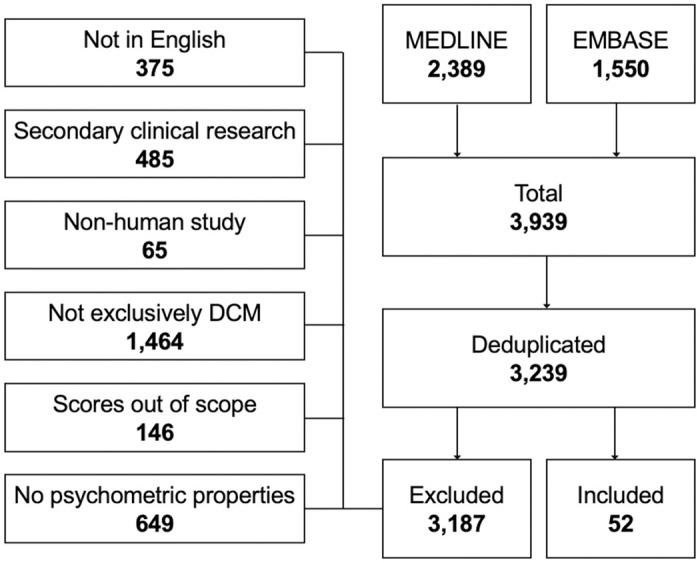
PRISMA Flow Diagram, CMS systematic review. CSM, cervical spondylotic myelopathy; DCM, degenerative cervical myelopathy.

The primary literature search identified a total of 3,239 unduplicated studies (MEDLINE: 2,389, EMBASE: 1,550). From this search, 52 met eligibility criteria (Fig 4) and consisted of 7,395 patients and 29 instruments.

**Fig 5:**
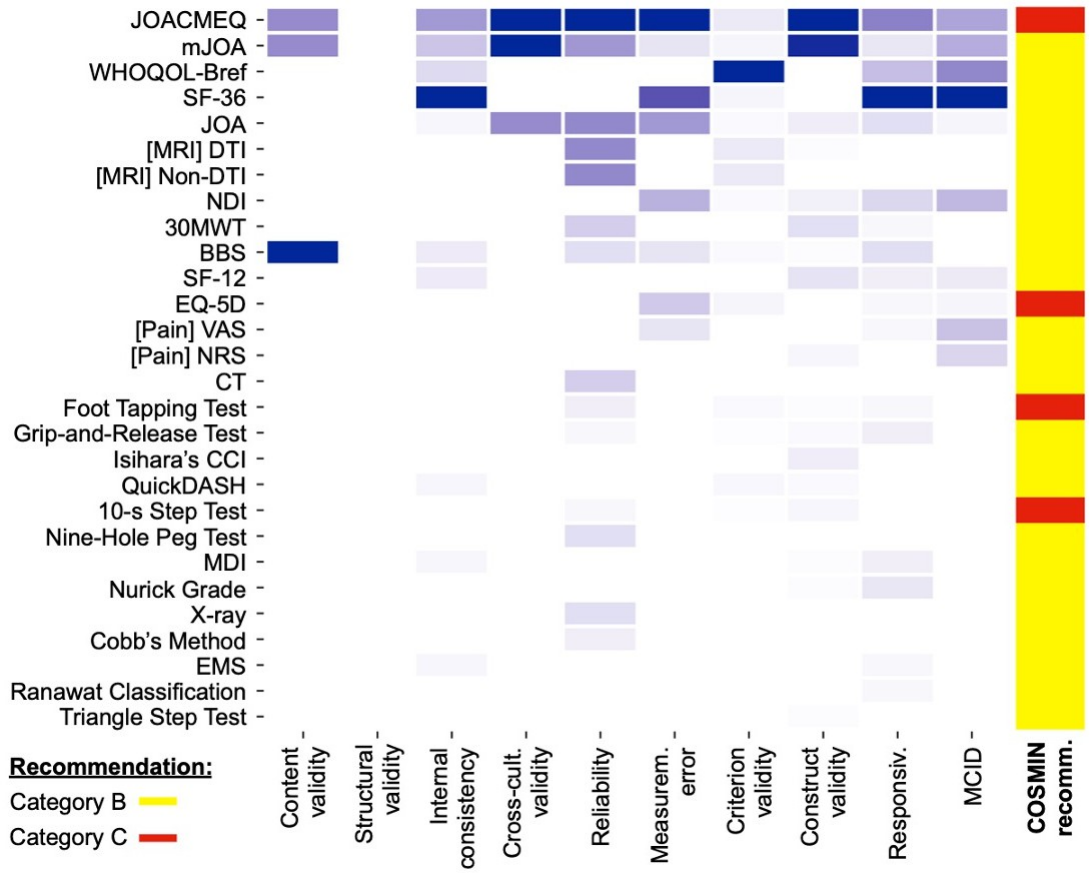
Number of studies for each outcome measure and property [column-normalised]. Shades of purple represent the number of studies per clinimetric property per outcome measure. Included studies reported on at least one of the 10 COSMIN properties for all instruments. No instrument had evidence for all 10 properties and <50% (13/29) of instruments had evidence for at least one property per measurement domain. Notably, no instruments were evaluated for structural validity, attained sufficient evidence for content validity, or obtained a Category A recommendation based on COSMIN criteria. (COSMIN recommendation categories: A = measurement instruments with evidence for sufficient content validity (any level) AND at least low-quality evidence for sufficient internal consistency; B = measurement instruments categorised not in A or C; C = measurement instruments with high-quality evidence for an insufficient measurement property.)

The measurement properties of the 29 instruments were evaluated using the COSMIN methodology for systematic reviews. A summary of findings is presented in Table 4: (1) the overall feasibility rating; (2) the overall interpretability rating; and (3) the overall recommendation category based on existing evidence. Included studies reported on at least one of the 10 COSMIN properties for all instruments. No instrument had evidence for all 10 properties and <50% (13/29) of instruments had evidence for at least 1 property per measurement domain (Fig 5).

**Table 4:** A summary of findings.

Domain	Instrument	Feasibility	Interpretability	Recommendation category
Life impact	EQ-5D	+	+	C
	SF-12	–	+	B
	SF-36	–	+	B
	WHOQOL-Bref	+	–	B
Life impact and neurological function	JOACMEQ	+	+	B
Neurological function	10 s step test	+	–	C
	30MWT	+	–	C
	9-Hole peg test	++	–	B
	BBS	++	–	B
	European Myelopathy Scale	+	–	B
	Foot tapping test	+	–	C
	Grip-and-release test	+	–	B
	JOA	–	+	B
	MDI	+	–	B
	mJOA	–	+	C
	Nurick scale	+	–	B
	P-mJOA	+	–	B
	Ranawat classification of disease severity	–	–	B
	Triangle step test	+	–	B
Pain and neurological function	QuickDASH	–	–	
Pain	NDI	+	+	B
	Arm pain score	–	+	B
	Neck pain score	+	+	B
	VAS for pain	+	+	B
Radiology	Cobb’s method	+	–	B
	CT (Tsuyama’s classification, 2D and 3D)	+	–	B
	CT (Tsuyama’s classification, lateral + axial)	+	–	B
	Isihara’s cervical curvature index	+	–	B
	MRI (depiction of intramedullary hyperintensity at eight cervical disc levels, T2W, 1.5-T or 3-T)	+	–	B
	MRI (Kang’s classification, 1.5-T or 3-T)	+	–	B
	MRI (Muhle’s classification, 1.5-T)	+	–	B
	MRI (Vaccaro’s classification, 1.5-T)	+	–	B
	X-rays (computer-assisted measurement of length and thickness)	+	–	B

Feasibility: ++ = No barriers; + = Minimal barriers;– = Barriers.

Interpretability:+ = Interpretable;– = Uninterpretable, due to absence of anchor-based MCIDs.

Recommendation category: A = measurement instruments with evidence for sufficient content validity (any level) AND at least low-quality evidence for sufficient internal consistency; B = measurement instruments categorised not in A or C; C = measurement instruments with high-quality evidence for an insufficient measurement property.

BBS, Berg Balance Scale; EQ-5D, EuroQol-5 Dimension; JOA, Japanese Orthopaedic Association; JOACMEQ, Japanese Orthopaedic Association Cervical Myelopathy Evaluation Questionnaire; MDI, Myelopathy Disability Index; mJOA, modified Japanese Orthopaedic Association; 30MWT, 30-m Walking Test; NDI, Neck Disability Index; P-mJOA, patient-derived version of the mJOA; SF-12, 12-Item Short Form Health Survey; SF-36, 36-Item Short Form Health Survey; VAS, Visual Analogue Scale; WHOQOL-Bref, World Health Organisation Quality of Life.

No category A recommendations were made as no measurement instrument had sufficient evidence for content validity. Next, due to the availability of high-quality evidence for insufficient criterion validity, construct validity, and/or responsiveness, 4 instruments were recommended for category C. However, most instruments were categorised into category B due to the notable absence of high-quality evidence in most measurement properties.

Considering these results and given both (1) the very strict quality standards of the COSMIN framework; and (2) that the absence of evidence is not the same as poor-quality evidence, it was agreed that instruments most suitable for use should be interpretable by clinicians and offer qualitative meaning to either clinicians or patients. To this end, the measurement properties of the 9 interpretable instruments are presented in Table 5: the arm and neck pain scores; SF-12 and SF-36; JOA, mJOA, and JOACMEQ; NDI; and VAS for pain. These include 1 score with insufficient criterion and construct validity (i.e., mJOA) and 6 scores with barriers to application.

**Table 5:** Instruments identified from the CMS Systematic Review, meeting the recommendation shortlist.

Domain	Instrument	Psychometric properties*	Feasibility	Recommendation category
Life impact				
	SF-12	Cronbach’s α coefficient (0.77)	–	B
	MCS	SCB (51.5)		
	PCS	SCB (30.1) Responsiveness: SF-12 PCS (Mean change score: 8.17)		
	SF-36	Cronbach’s α coefficient (0.79–0.93) Responsiveness: SF-36 (Normalised change: 0.32)	–	B
	MCS	MDC or SDC (Distribution: 3.3–5.7) MCID (Distribution: 3.4–6.8, anchor: 3.0–7.4) Construct validity: Arm pain score (Pearson’s correlation: –0.23) mJOA scale (Pearson’s correlation: 0.19) NDI (Spearman’s rank correlation: –0.17) Neck pain score (Pearson’s correlation: –0.28) SF-12 PCS (Pearson’s correlation: 0.01) Responsiveness: SF-36 MCS (Effect size range: 0.81, sensitivity: 0.67)		
	PCS	MDC or SDC (Distribution: 5.2–5.7, anchor: 4.9) MCID (Distribution: 2.9–5.5, distribution: 10, anchor: 3.9–9.6) SCB (16) Criterion validity (Likert scale) AUC: 0.67–0.69 Construct validity: Arm pain score (Pearson’s correlation: –0.44) mJOA scale (Pearson’s correlation: 0.43) NDI (Spearman’s rank correlation: –0.49) Neck pain score (Pearson’s correlation: –0.41) SF-12 PCS (Pearson’s correlation: –0.29) Responsiveness: SF-36 PCS (Effect size range: 0.84, sensitivity: 0.85)		
Life impact and neurological function				
	JOACMEQ	Patient comprehensibility: “No questions elicited no answer or “I am not sure” in more than 5% of patients” Test–retest stability: Cronbach’s α coefficient (0.91) Forward-backward translation [Persian and Thai]: n/a	+	B
	Bladder function	Cronbach’s α coefficient (0.32–0.74) Test–retest stability: ICC (0.62) MDC or SDC (Distribution: 7.7) MCID (Anchor: 6.0) Responsiveness: JOACMEQ Bladder function (AUC: 0.82, Effect size: 0.33, Mean change score: 18.0)		
	Cervical spine function	Cronbach’s α coefficient (0.77–0.78) Test–retest stability: ICC (0.63) MDC or SDC (Distribution: 12.9, anchor: 12.5) MCID (Anchor: 2.5) Criterion validity (Likert scale) AUC: 0.58 Responsiveness: JOACMEQ Cervical spine function (AUC: 0.72, Effect size: 0.28, Mean change score: 25.8)		
	Lower extremity function	Cronbach’s α coefficient (0.80–0.86) Test–retest stability: ICC (0.83) MDC or SDC (Distribution: 6.6, anchor: 8.5) MCID (Anchor: 8.5–9.5) Criterion validity (Likert scale) AUC: 0.66–0.70 Construct validity: NDI (Pearson’s correlation: –0.66) SF-12 MCS (Spearman’s rank correlation: 0.40) SF-12 PCS (Spearman’s rank correlation: 0.29) Responsiveness: JOACMEQ Quality of life (AUC: 0.83, Effect size: 0.46, Mean change score: 23.7)		
	QOL	Cronbach’s α coefficient (0.80–0.86) Test–retest stability: ICC (0.83) MDC or SDC (Distribution: 6.6, anchor: 8.5) MCID (Anchor: 8.5–9.5) Criterion validity (Likert scale) AUC: 0.66–0.70 Construct validity: NDI (Pearson’s correlation: –0.66) SF-12 MCS (Spearman’s rank correlation: 0.40) SF-12 PCS (Spearman’s rank correlation: 0.29) Responsiveness: JOACMEQ Quality of life (AUC: 0.83, Effect size: 0.46, Mean change score: 23.7)		
	Upper extremity function	Cronbach’s α coefficient (0.72–0.74) Test–retest stability: ICC (0.93) MDC or SDC (Distribution: 9.5, anchor: 6.1) MCID (Anchor: 2.5–13.0) Responsiveness: JOACMEQ Upper extremity function (AUC: 0.74, Effect size: 0.17, Mean change score: 10.7)		
Neurological function				
	JOA	Cronbach’s α coefficient (0.72) Forward-backward translation [Brazilian Portuguese]: Comprehension rate (>81.2%) Inter-observer reliability: ICC (0.81) MDC or SDC (Distribution: 1.0, anchor: 2.5) LOA (1.2 [–1.2, 3.6]) MCID (Anchor: 2.5) Criterion validity (Likert scale) AUC: 0.59–0.62 Construct validity: JOACMEQ QOL (Spearman’s rank correlation: 0.41) mJOA (Spearman’s rank correlation: 0.87) NDI (Spearman’s rank correlation: –0.50 to –0.76) SF-12 MCS (Spearman’s rank correlation: –0.05) SF-12 PCS (Spearman’s rank correlation: 0.50) Responsiveness: JOA (Mean change score: 4.6, normalised change: 0.21) JOA Motor function of lower extremity (Mean change score: 0.60) mJOA (Spearman’s rank correlation: 0.75)	–	B
	Bladder function	Intra-observer reliability (κ = 0.64) Inter-observer reliability (κ = 0.47)		
	Motor function of fingers	Intra-observer reliability (κ = 0.68) Inter-observer reliability (κ = 0.53)		
	Motor function of shoulder and elbow	Intra-observer reliability (κ = 0.50) Inter-observer reliability (κ = 0.31)		
	Motor function of lower extremity	Intra-observer reliability (κ = 0.55) Inter-observer reliability (κ = 0.49)		
	Sensory function of lower extremity	Intra-observer reliability (κ = 0.54) Inter-observer reliability (κ = 0.58)		
	Sensory function of upper extremity	Intra-observer reliability (κ = 0.51) Inter-observer reliability (κ = 0.42)		
	mJOA	Cronbach’s α coefficient (0.60–0.63) Forward-backward translation [Brazilian Portuguese and Italian]: n/a Test–retest stability (Spearman’s rank correlation: 0.91) Intra-observer reliability (ICC: 0.87) Inter-observer reliability (ICC: 0.97, κ = 0.80) MDC or SDC (Distribution: 2.1) MCID (Distribution: 1.2–1.4, anchor: 1.3–3.1) SCB (14) Criterion validity (Nurick scale) Spearman’s rank correlation: –0.41, Pearson’s correlation: –0.62 to –0.63 Construct validity: 30MWT (Pearson’s correlation: –0.38) EQ-5D (Spearman’s rank correlation: 0.42) JOACMEQ QOL (Spearman’s rank correlation: 0.41) NDI (Spearman’s rank correlation: –0.51, Pearson’s correlation: –0.33 to –0.34) SF-12 MCS (Pearson’s correlation: 0.03) SF-12 PCS (Pearson’s correlation: 0.42) SF-36 MCS (Pearson’s correlation: 0.25) SF-36 PCS (Pearson’s correlation: 0.30) Responsiveness: mJOA (Effect size: 0.87–1.0, normalised change: 1.47)	–	C
	Motor dysfunction of lower extremities	Inter-observer reliability (ICC: 0.73) Criterion validity (Nurick scale) Pearson’s correlation: –0.65 to –0.68 Construct validity: 30MWT (Pearson’s correlation: –0.43) NDI (Pearson’s correlation: –0.31) SF-36 MCS (Pearson’s correlation: 0.21) SF-36 PCS (Pearson’s correlation: 0.31–0.50)		
	Motor dysfunction of upper extremities	Inter-observer reliability (ICC: 0.77) Criterion validity (Nurick scale) Pearson’s correlation: –0.42 Construct validity: 30MWT (Pearson’s correlation: –0.21) NDI (Pearson’s correlation: –0.24) SF-36 MCS (Pearson’s correlation: 0.20) SF-36 PCS (Pearson’s correlation: 0.22)		
	Sensory dysfunction of sphincter dysfunction	Inter-observer reliability (ICC: 0.78) Criterion validity (Nurick scale) Pearson’s correlation: –0.25 Construct validity: 30MWT (Pearson’s correlation: –0.23) NDI (Pearson’s correlation: –0.16) SF-36 MCS (Pearson’s correlation: 0.08) SF-36 PCS (Pearson’s correlation: 0.06)		
	Sensory dysfunction of upper extremities	Inter-observer reliability (ICC: 0.93) Criterion validity (Nurick scale) Pearson’s correlation: –0.23 Construct validity: 30MWT (Pearson’s correlation: –0.05) NDI (Pearson’s correlation: –0.23) SF-36 MCS (Pearson’s correlation: 0.19) SF-36 PCS (Pearson’s correlation: 0.19)		
Pain				
	NDI	MDC or SDC (Distribution: 6.2%, anchor: 5.2%) MCID (Anchor: 5–13) SCB (Anchor: 9.5–36) Criterion validity (Likert scale) AUC: 0.66–0.75 Construct validity: Arm pain score (Pearson’s correlation: 0.68) mJOA (Pearson’s correlation: –0.36) Neck pain score (Pearson’s correlation: 0.64) SF-12 MCS (Pearson’s correlation: –0.40) SF-12 PCS (Pearson’s correlation: –0.54) Responsiveness: Anchor (AUC: 0.66) NDI (Mean change score: –15.8)	+	B
	Pain, “Numeric rating scale” (arm pain score)	MCID (Anchor: 2.5) SCB (3.5) Construct validity: mJOA (Pearson’s correlation: –0.19) Neck pain score (Pearson’s correlation: 0.72)	–	B
	Pain, “Numeric rating scale” (neck pain scores)	MCID (Anchor: 2.5) SCB (3.5) Construct validity: mJOA (Pearson’s correlation: –0.07)	–	B
	VAS for pain	MDC or SDC (Distribution: 3.1) MCID (Distribution: 24.0–30.0, anchor: 0.4–2.7) SCB (1.1)	+	B

CMS, core measurement set; MCID, minimally clinical important difference; mJOA, modified Japanese Orthopaedic Association score; SCB, substantial clinical benefits.

#### Gap analysis

While the review identified clinically interpretable instruments that were common to DCM research and could be used to measure outcomes in the COS, there were: (1) several outcomes for which no existing instrument was appropriate; and (2) several instruments for which the evidence base was deemed inadequate [47].

The initial search of MEDLINE did not identify any suitable scoping reviews for alternative instruments but did identify the protocol for one suitable scoping review for fatigue. The results of this were obtained via personal communication. Following input from the SC, outcomes within the domain of pain were excluded as it was felt the resources and recommendations aggregated by the Initiative on Methods, Measurement and Pain Assessment in Clinical Trials (IMMPACT) were sufficient [48]. The remaining gaps underwent the targeted scoping review and shortlisting procedure (Table 6), followed by COSMIN evaluation.

**Table 6:** Gap analysis, scoping, and shortlist for alternative instruments. Elements with at least 1 interpretable instrument (see Phase 2.1) are shaded. Targeted searches of MEDLINE were performed for the remaining elements (i.e., “gaps,” unshaded, see Phase 2.2). For gaps within the domain of pain (hashed), the resources aggregated by IMMPACT were deemed sufficient [48]. The number of articles (N) screened is indicated for each gap. Notably, only 1 suitable resource was identified for “fatigue.”

Domain	Outcome	Gap analysis	Shortlisted candidate instruments
Adverse events	Death		
	Surgical adverse events	0 (*N* = 55)	Spine adverse events severity system, version 2 (SAVES2)
Economic impact	Cost of care		
	Employment status	0 (*N* = 5)	Sydney psychosocial reintegration scale (SPRS) Valuation of lost productivity (VOLP)
Life impact	Dependence		
	Falls	0 (*N* = 173)	Activities-specific balance confidence (ABC) scale Falls efficacy scale (FES)
	Fatigue	1 (*N* = 207)	Fatigue assessment instrument (FAI) Functional assessment of chronic illness therapy-fatigue (FACIT-F) scale
	Mental health		
	Mobility		
Neurological function	Arm strength		
	Balance		
	Bladder function		
	Faecal incontinence	0 (*N* = 308)	Faecal incontinence questionnaire (FIQ) Wexner score
	Dexterity		
	Finger strength		
	Grip strength		
	Leg strength		
	Muscle tone and spasticity	0 (*N* = 39)	Ashworth scale Modified Ashworth scale
	Neck mobility		
	Sensation		
Pain	Location		
	Intensity		
	Pain control		
	Perception		
Radiology	Adjacent segment degeneration	0 (*N* = 69)	Disc degeneration Hilibrand’s criteria Kellgren–Lawrence New spinal canal stenosis
	Cervical spine alignment	0 (*N* = 24)	C2-C7 Cobb angle C2-C7 sagittal vertical axis T1 slope
	Cord compression	0 (*N* = 69)	AP diameter CSA Matsumoto et al
	Cord signal change	0 (*N* = 24)	RC length of signal change Sagittal T1WI signal hypointensity Sagittal Type I-III T2WI signal hyperintensities

#### Stage 3

A face-to-face consensus meeting was held alongside the Global Spine Congress, 2022 in Las Vegas. A mixed group of stakeholders, including PwDCM, Spine Surgeons, a Neurologist, Physiotherapists, and a clinical trial statistician attended (Table 7). The predefined make-up of the panel was considered to have sufficiently met the a priori criteria; specifically all expertise was represented, only half were spinal surgeons, 5 out of the 12 members were external to the SC, and 2 out of 12 PwDCM. Only 3 out of 12 identified as female.

**Table 7:** CMS consensus meeting participants.

Name	Profession	Location
Dr. Michael Fehlings	Neurosurgeon	Canada
Dr. James Guest	Neurosurgeon	USA
Dr. Rory Murphy	Neurosurgeon	USA
Dr. Allan Martin	Neurosurgeon	USA
Dr. Mark Kotter	Neurosurgeon	UK
Dr Brian Kwon	Orthopaedic Surgeon	Canada
Dr Lindsay Tetreault	Neurologist	USA
Dr David Anderson	Physiotherapist	Australia
Dr Nader Fallah	Trial Statistician	Canada
Tammy Blizzard	PwDCM	USA
Timothy Boerger	PwDCM and Clinical Researcher	USA
Caroline Treanor	Physiotherapist	Ireland

Using facilitated discussion and the aggregate results of the pre-meeting survey, final consensus recommendations were made (Table 8). For Radiology, however, no instrument was selected as it was felt the current measurement options did not fulfil the criteria of an “outcome” measure. It was instead felt to represent a sampling characteristic (data element).

**Table 8:** Final consensus recommendations core measurement set.

Domain	Shortlisted instruments	Final consensus
Pain	NDI SF-MPQ Pain NRS (neck) Pain NRS (whole body) Pain NRS (arm) VAS for pain	NDI
Neurological function	mJOA JOACMEQ 9-hole peg test 6-, 10-m walk test GRASSP-M 30m walk test	mJOA/JOA
Adverse events	SAVES2 Clavien-Dindo Classification	SAVES2
Life Impact	SF-12 SF-36 ABC Scale JOACMEQ EQ-5D-5L FACIT-F	SF-36 (MCS and PCS)
Economic impact	SPRS VOLP	(Based on SF-36)
Radiology	CSA Sagittal Type I-II T2WI signal hyperintensities C2-C7 Cobb Angle sagittal C2-C7 Sagittal Vertical Axis	

For the mJOA, the version proposed by Benzel and colleagues was recommended [49].

A presentation summarising the recovery profiles observed in the preselected data sets was provided (AM). The AO Spine studies conducted follow up after surgery at 6, 12, and 24 months, whereas CSORN assessed patients at 3, 12, and 24 months. Data demonstrated that the most meaningful recovery (defined using the mJOA) occurred by the first follow up in each study, with no significant change between 12 and 24 months. However, from these data sets, a comparison of 3 versus 6 months was not possible. Consensus favoured selecting an early time point (3 or 6 months) for standardisation, to avoid imposing the implications of long-term follow-up with apparent diminishing returns for cohort separation. The consensus group eventually recommended follow-up assessment to include 6 months. This principally recognised 6 months as the more popular existing timepoint (compared to 3 months), less likely to be influenced by reversible surgical adverse events, and likely to capture more meaningful recovery.

#### Formation of clinical research forms (CRFs)

The data set was implemented into CRF to support implementation. These were based on the experience of authors in the delivery of 2 current DCM trials: POLYFIX-DCM ISRCTN12638817 and RECEDE-Myelopathy ISRCTN16682024. CRFs were prepared for baseline, surgical admission, and postsurgical follow-up (Supplementary 5). Deformity was included as a CDE, recognising that it was originally included as a CDE, but then dropped as it was also selected as an outcome but ultimately not included in the final CMS.

## Discussion

Using extensive input from the literature, and a global multidisciplinary community including people living with DCM, recommendations were developed on what to report as a minimum in clinical trials of DCM, including how and when outcomes should be measured.

In total, 28 outcomes and 6 domains (Pain, Neurological Function, Life Impact, Radiology, Economic Impact, and Adverse Events) were entered into the final COS. Thirty two outcomes and 4 domains (Individual, Disease, Investigation, and Intervention) were entered into the final CDE. Finally, 4 outcome instruments (mJOA, NDI, SF-36v2, SAVES2) were identified for the CMS, with a recommendation for trials evaluating outcomes after surgery, to include baseline measurement and at 6 months from surgery.

### A pragmatic framework, with strong foundations to evolve over time and support research targeting the leading research priorities

Acknowledging that the standardisation of data measurement and reporting is an immediate priority for DCM, it was decided that the initial CMS should focus on selecting the most relevant—but existing—instruments, as opposed to developing new tools or selecting those early in development. The focus on existing instruments in use has overlooked those in development, those published in languages other than English and those at early stages of adoption. The selection of one instrument per domain has also potentially overlooked core outcomes. Further, the scoping approach outside of DCM was pragmatic, with a cursory assessment of content validity as the principal yardstick of applicability. While this risked missing relevant tools or using tools of unknown quality in DCM, we suspect this is very unlikely to limit the CMS. First, the shortlisting used a systematic and structured approach, adapted from the prioritization of databases and standards in the COSMIN website and manual (respectively) [10–12]. The rigour of the COSMIN criteria means that tools at early stages of development rarely meet the threshold for inclusion [23]. Secondly, the process incorporated opportunities for expert opinion/recommendations, including PwCM to be integrated. However finally, and importantly, it has produced what appears to those involved, to be a readily implementable set. The goal of a CMS is to enable standardisation across trials. For this to happen it needs to be concise. Based on present measurement tools, a CMS covering all core outcomes would have been a burden for trials. It is likely this CMS will need to be updated, preferably with a dedicated and comprehensive tool. For now, this represents a practical collection of measures, with broad relevance to the COS and one that is readily benchmarked against leading evidence from the last decade [50–54].

Within the minimum data set, there is notable synergy with the DCM research priorities. For example, the inclusion of economic outcomes in the data set coincides with the research priority of establishing the socioeconomic impact of DCM [6, 27]. A second example is the inclusion of “ethnicity,” directly referenced by stakeholders to align with the priority of identifying genetic determinants in DCM [15,18,55,56]. We hypothesise that this synergy has arisen as the minimum data set was developed alongside a James Lind Alliance research priority setting partnership. To our knowledge, this is the first example of a combined approach and may ensure that the DCM minimum data set can not only drive standardisation of research, but act as a knowledge translation intervention to ensure further research targets key research priorities.

Another novel aspect of this process was the decision to make a recommendation on “when” to report outcomes after surgical treatment. To be clear, this is not intended to define a trials primary endpoint but simply ensure that there is at least 1 consistent time point across studies where outcomes are reported. This will be important for many DCM scenarios, where recovery after surgery is a function of time since surgical intervention [54].

### Effective and sustained implementation now essential for impact

Standardisation initiatives are only as effective as their implementation [57,58]. An exemplar from minimum data set research is seen for rheumatoid arthritis, where increased adoption has paralleled a transformation in patient outcomes [59]. The importance of implementation has become more and more pertinent throughout the AO Spine RECODE-DCM, as referenced by the selection of “Raising Awareness” as the leading research priority [11] and efforts to sustain the network that the project formed [23].

For this reason, many pragmatic decisions were taken during this process, such as categorising the CMS into domains (rather than stipulated outcomes) and using tools familiar to the research community. Further, the preparation of template research documents, as part of this publication, represents a novel effort to facilitate adoption. Nevertheless, the DCM research field is small and fragmented [60], with many examples of siloed knowledge. Ensuring the tools created by AO Spine RECODE-DCM, including the minimum data set, can accelerate progress will require a sustained and community wide effort.

This effort will need to be more than simply promoting adoption. Effective implementation, or knowledge translation, is characterised by a cycle [61] that must continually identify and adapt to change as it occurs within the landscape it targets. DCM research to date has focused on surgical treatment and/or moderate to severe disease [62,63]. This experience will be reflected in the consensus processes, both from the evidence scoped, but also the experience that stakeholders can draw upon. The potential need for the data set to evolve, as new instruments arise, is already mentioned, but it may also need to adapt as specific research themes increase and/or more selective inclusion criteria. For example, the onset of DCM from Asymptomatic Spinal Cord Compression is now a critical research priority, with limited representation in the foundational steps of this process (e.g., evidence reviews, or PwDCM). The applicability of this data set to this research setting is therefore uncertain. One could argue that the scope should have been narrowed at the start. We would argue for a field so small and disconnected, we should start with unity around a single framework and evolve when and if it becomes necessary. This is very much how OMERACT started [64], evolving from 1 data set for rheumatoid arthritis 30 years ago, to include subspecialised sets, for example related to imaging [65,66].

### Conclusions

DCM is a progressive chronic spinal cord injury. Without standardised guidance, clinical research studies have selected outcomes at their discretion, often underrepresenting the disease and limiting comparability between studies.

AO Spine RECODE-DCM has produced a minimum data set for use in DCM clinical trials today. While it is anticipated the CDE and COS will have strong and durable relevance, it is acknowledged that new measurement tools, alongside an increasing transition to study patients not undergoing surgery, may necessitate updates and adaptation, particularly with respect to the CMS.

## Supporting information

S1 Data COS STAR Reporting Checklist. (DOCX)

S2 Data AO Spine RECODE-DCM Steering Committee Members: Name and Affiliation, including represented stakeholder group. (DOCX)

S3 Data AO Spine RECODE-DCM Management Group, responsible for day-to-day project management and support. (DOCX)

S4 Data Detailed sampling characteristics for respondents in the Round 1 COS survey. For categorical data (e.g., Gender or Country of Residence), values are count and proportions. For continuous data (e.g., Age), values are mean ± standard deviation for normally distributed data. The exception is years since diagnosis, for which data was skewed, and is represented as median ± inter quartile range (IQR).
(DOCX)

S5 Data Template clinical research forms. (PDF)

## Abbreviations

CDE: core data elements (or comment data element)
ClinROM: clinician reported outcome measure
CMS: core measurement set
COMET: Core Outcome Measures in Effectiveness Trials
COS: core outcome set (or comment outcome set)
COSMIN: Consensus-based Standards for the selection of health Measurement Instruments
COS-STAR: Core Outcome Set-STAndards for Reporting
CRF: clinical research form
CSM: cervical spondylotic myelopathy
CSORN: Canadian Spine Outcomes and Research Network
DCM: degenerative cervical myelopathy
GRADE: Grading of Recommendations, Assessment, Development and Evaluations
IMMPACT: Initiative on Methods, Measurement and Pain Assessment in Clinical Trials
MCID: minimally clinical important difference
mJOA: modified Japanese Orthopaedic Association score
oHCP: other Healthcare Professionals
OMERACT: outcomes measurement in rheumatology
OML: Outcomes Measures Library
OPLL: ossification of the posterior longitudinal ligament
PROM: patient reported outcome measure
PwCM: patients with DCM
SC: steering committee
SCB: substantial clinical benefits
