# Supplementary material for: A minimum data set—Core outcome set, core data elements, and core measurement set—For degenerative cervical myelopathy research (AO Spine RECODE DCM): A consensus study
Source: PLoS Med. 2024 Aug 22;21(8):e1004447. doi: 10.1371/journal.pmed.1004447 (PMC11379399; doi:10.1371/journal.pmed.1004447)
Supplement: S2 Data — (DOCX) [file pmed.1004447.s002.docx]

Supplementary Data 2 AO Spine RECODE-DCM Steering Committee Members: Name and Affiliation, including represented stakeholder group

| **Name** | **Role** | **Stakeholder Group** | **Country** |
| --- | --- | --- | --- |
| Evangeline Howard | Person with DCM | Lived Experience | USA |
| Iwan Sadler | Person with DCM | Lived Experience | UK |
| Ellen Sarewitz | Person with DCM | Lived Experience | UK |
| Delphine Houlton | Person with lived experience | Lived Experience | UK |
| Julia Carter | Person with DCM | Lived Experience | UK |
| Margot Miller | Person with DCM | Lived Experience | USA |
| Theresa Brislin | Person with DCM | Lived Experience | USA |
| Timothy Boerger | Person with DCM | Lived Experience | USA |
| Carla Salzman | Person with DCM | Lived Experience | USA |
| Jillian Polasik | Person with DCM | Lived Experience | UK |
| Shirley Widdop | Person with DCM | Lived Experience | UK |
| Armin Curt | Neurologist/Neurorehabilitation | Other Healthcare Professional | Switzerland |
| Sukhvinder Kalsi-Ryan | Physiotherapist | Other Healthcare Professional | Canada |
| Anoushka Sing | Nurse | Other Healthcare Professional | Canada |
| Julio Furlan | Neurologist/Neurorehabilitation | Other Healthcare Professional | Canada |
| Chen Robert | Neurologist/Neurorehabilitation | Other Healthcare Professional | Canada |
| Katherine Palmieri | Anaesthesiologist | Other Healthcare Professional | USA |
| Geno J. Merli | Pain Medicine | Other Healthcare Professional | USA |
| James Milligan | Family Physician | Other Healthcare Professional | Canada |
| Michelle Starkey | Director Myelopathy.org | Scientist | UK |
| Michael Fehlings | Neurosurgeon | Surgeon | Canada |
| Brian Kwon | Orthopaedic Surgeon | Surgeon | Canada |
| Shekar Kurpad | Neurosurgeon | Surgeon | USA |
| Bizhan Aarabi | Neurosurgeon | Surgeon | USA |
| Vafa Rahimi Movaghar | Neurosurgeon | Surgeon | Iran |
| James Harrop | Neurosurgeon | Surgeon | USA |
| James Guest | Neurosurgeon | Surgeon | USA |
| Mark Kotter | Neurosurgeon | Surgeon | UK |
| Benjamin M. Davies | Neurosurgeon | Surgeon | UK |
| Jefferson R Wilson | Neurosurgeon | Surgeon | Canada |
| Ricardo Rodrigues-Pinto | Orthopaedic Surgeon | Surgeon | Portugal |
