# Supplementary material for: A minimum data set—Core outcome set, core data elements, and core measurement set—For degenerative cervical myelopathy research (AO Spine RECODE DCM): A consensus study
Source: PLoS Med. 2024 Aug 22;21(8):e1004447. doi: 10.1371/journal.pmed.1004447 (PMC11379399; doi:10.1371/journal.pmed.1004447)
Supplement: S3 Data — (DOCX) [file pmed.1004447.s003.docx]

Supplementary Data 3 AO Spine RECODE-DCM Management Group, responsible for day-to-day project management and support.

| **Name** | **Role** | **Stakeholder Group** |
| --- | --- | --- |
| Mark Kotter | Principal Investigator | Surgeon |
| Benjamin Davies | Principal Investigator | Surgeon |
| Iwan Sadler | Person with lived experience | Lived Experience |
| Ellen Sarewitz | Person with lived experience | Lived Experience |
| Olesja Hazenbiller | AO Spine Project Coordinator | N/A |
| Lindsay Tetreault | Information Specialist | Other Healthcare Professional |
| Danyal Khan | Clinical Researcher | N/A |
| Oliver Mowforth | Clinical Researcher | N/A |
