# Supplementary material for: A minimum data set—Core outcome set, core data elements, and core measurement set—For degenerative cervical myelopathy research (AO Spine RECODE DCM): A consensus study
Source: PLoS Med. 2024 Aug 22;21(8):e1004447. doi: 10.1371/journal.pmed.1004447 (PMC11379399; doi:10.1371/journal.pmed.1004447)
Supplement: S4 Data — For categorical data (e.g., Gender or Country of Residence), values are count and proportions. For continuous data (e.g., Age), values are mean ± standard deviation for normally distributed data. The exception is years since diagnosis, for which data was skewed, and is represented as median ± inter quartile range (IQR). (DOCX) [file pmed.1004447.s004.docx]

Supplementary Data 4 Detailed sampling characteristics for respondents in the Round 1 COS survey. For categorical data (e.g. Gender or Country of Residence), vales are count and proportions. For continuous data (e.g. Age), values are mean ± Standard Deviation for normally distributed data. The exception is years since diagnosis, for which data was skewed, and is represented as median ± Inter Quartile Range (IQR).

| **Demographic** | **COS, N(%) or Average (±Variation)*** |
| --- | --- |
| **Person with DCM** | **n=113** |
| **Age** | 56.6 +/-9.4 |
| **Male** | 29 (25.7) |
| **Country of residence** | |
| United States | 30 (28.6) |
| United Kingdom | 37 (35.2) |
| Uganda | 11 (10.5) |
| Ecuador | 3 (2.9) |
| Ukraine | 7 (6.7) |
| United Arab Emirates | 7 (6.7) |
| Norway | 1 (1.0) |
| Netherlands | 2 (1.9) |
| Other | 5 (5.0) |
| **Years since diagnosis** | 3.5 (IQR 5) |
| **Received Surgery** | 85 (75.2) |
| **Received Physiotherapy** | 71 (62.8) |
| **mJOA score** | 12.0 +/- 2.6) |
| **Pain score** | 5.6 +/- 2.2 |
| **Employment** | |
| Full-time employment | 24 (21.2) |
| Part-time employment | 17 (15.0) |
| Unemployed | 8 (7.1) |
| Unable to work | 38 (33.6) |
| Retired | 26 (23.0) |
| **Spinal Surgeons** | **n=158** |
| **Age** | 44.6 +/-10.2 |
| **Male** | 152 (96.2) |
| **Country of practice** | |
| United States | 16 (10.1) |
| India | 17 (10.8) |
| United Kingdom | 16 (10.1) |
| Canada | 7 (4.4) |
| Japan | 8 (5.1) |
| Italy | 11 (7.0) |
| Australia | 7 (4.4) |
| Germany | 6 (3.8) |
| Portugal | 1 (0.6) |
| Greece | 3 (1.9) |
| South Korea | 2 (1.3) |
| Ukraine | 1 (0.6) |
| Other | 63 (39.9) |
| **Training speciality** | |
| Neurosurgery | 89 (56.3) |
| Orthopaedics | 69 (43.7) |
| **Years managing DCM** | 13.2 +/-8.5 |
| **DCM patients per year** | |
| 0-25 | 37 (23.4) |
| 25-50 | 51 (32.3) |
| 50-100 | 39 (24.7) |
| 100+ | 31 (19.6) |
| **Other Healthcare Professionals** | **n=61** |
| **Age** | 42.9 +/-10.2 |
| **Male** | 38 (62.3) |
| **Country of practice** | |
| United States | 6 (10.0) |
| Canada | 15 (25.0) |
| United Kingdom | 7 (11.7) |
| Ireland | 2 (3.3) |
| Italy | 2 (3.3) |
| Australia | 8 (13.2) |
| Switzerland | 3 (5.0) |
| United Arab Emirates | 3 (5.0) |
| Other | 13 (21.3) |
| **Profession** | |
| Family doctor/General Practitioner | 2 (3.3) |
| Neurologist | 7 (11.5) |
| Other allied health professional | 9 (14.8) |
| Other medical/surgical doctor | 17 (27.9) |
| Physiotherapist | 19 (31.1) |
| Researcher | 5 (8.2) |
| Specialist nurse | 2 (3.3) |
| **Years managing DCM** | 13.3 +/- 9.3 |
| **DCM patients per year** | |
| 0-25 | 34 (55.7) |
| 25-50 | 16 (26.2) |
| 50-100 | 9 (14.8) |
| 100+ | 2 (3.3) |
