# Supplementary material for: A minimum data set—Core outcome set, core data elements, and core measurement set—For degenerative cervical myelopathy research (AO Spine RECODE DCM): A consensus study
Source: PLoS Med. 2024 Aug 22;21(8):e1004447. doi: 10.1371/journal.pmed.1004447 (PMC11379399; doi:10.1371/journal.pmed.1004447)
Supplement: S5 Data — (PDF) [file pmed.1004447.s005.pdf]

Basic Information

Patient Details

Patient's Study Number: ☐☐☐ **Date of Birth:** M M / Y Y Y Y

This form was completed on: D D / M M / Y Y Y Y

Demographics

1. Ethnicity
   - ☐ Caucasian / White
   - ☐ Black
   - ☐ Asian
   - ☐ Hispanic
   - ☐ Other: ____________________
2. Biological gender ☐ Male ☐ Female
3. Age: ________ years old
4. Weight: ________ Kg
5. Height: ________ cm

Co-Morbidities

1. Psychiatric comorbidities ☐ Yes ☐ No

   1.1. If Yes, please state the name of the comorbidity ____________________
2. Diabetes ☐ Yes ☐ No
3. Obesity ☐ Yes ☐ No
4. Smoking status
   - ☐ Never smoked
   - ☐ Former smoker
   - ☐ Current smoker

   4.1. If current or former smoker, what is their pack year history?

   (Number of packs smoked per day multiplied by the number of years smoked for) __________

5. c
   - ☐ 0 Normal
   - ☐ 1 Symptomatic and ambulatory; cares for self
   - ☐ 2 Ambulatory >50% of time; occasional assistance
   - ☐ 3 Ambulatory <= 50% of time; nursing care needed
   - ☐ 4 Bedridden

Name and signature of person completing the form:		Date: D D / M M / Y Y Y Y

Basic Information

Patient Details

Patient's Study Number: ☐☐☐ **Date of Birth:** M M / Y Y Y Y

6. Other neurological disease ☐ Yes ☐ No
   6.1. If Yes, please state the name of the disease ____________________

DCM Characteristics

1. Date of first symptom(s): D D / M M / Y Y Y Y
2. Date of DCM Diagnosis: D D / M M / Y Y Y Y
3. Rate of progression ____________________
4. Number of previous surgery to treat DCM (times) ____________________
5. Co-existent radiculopathy ☐ Yes ☐ No

Investigation

1. Use of MRI imaging ☐ Yes ☐ No
2. Use of CT imaging ☐ Yes ☐ No
3. Level(s) of Compression

	**Anterior**	**Posterior**	**Circumferential**	**No compression**
3.1. C2 Vertebral Body	☐	☐	☐	☐
3.2. C2/C3 Intervertebral Disc	☐	☐	☐	☐
3.3. C3 Vertebral Body	☐	☐	☐	☐
3.4. C3/C4 Intervertebral Disc	☐	☐	☐	☐
3.5. C4 Vertebral Body	☐	☐	☐	☐
3.6. C4/C5 Intervertebral Disc	☐	☐	☐	☐
3.7. C5 Vertebral Body	☐	☐	☐	☐
3.8. C5/C6 Intervertebral Disc	☐	☐	☐	☐
3.9. C6 Vertebral Body	☐	☐	☐	☐
3.10. C6/C7 Intervertebral Disc	☐	☐	☐	☐
3.11. C7 Vertebral Body	☐	☐	☐	☐
3.12. C7/T1 Intervertebral Disc	☐	☐	☐	☐

4. MRI image findings: Presence of cord signal change

	**Yes**	**No**
4.1. T1 **Hypo**intensity	☐	☐
4.2. T2 **Hyper**intensity	☐	☐
4.3. Anterior Compression Only	☐	☐
4.4. Posterior Compression Only	☐	☐
4.5. Anterior and Posterior Compression	☐	☐

Name and signature of person completing the form: ____________________ Date: D D / M M / Y Y Y Y

Basic Information

Patient Details

Patient's Study Number: ☐☐☐ **Date of Birth:** M M / Y Y Y Y

5. Causative/Compressive pathology

	Yes	No
5.1. Spondylosis	☐	☐
5.2. Spondylosis with enlargement of ligamentum flavum	☐	☐
5.3. Spondylosis with OPLL	☐	☐
5.4. Disc pathology	☐	☐
5.5. OPLL	☐	☐
5.6. Spondylolisthesis	☐	☐
5.7. Klippel-Feil syndrome	☐	☐
5.8. Other (please state below if *Yes*):	☐	☐

6. Syrinx ☐ Yes ☐ No

7. Spondylolisthesis ☐ Yes ☐ No

8. Long tract signs ☐ Yes ☐ No

9. Radiological instability ☐ Yes ☐ No
(defined as subluxation on dynamic X-rays)

10. Alignment

☐ Lordosis

☐ Loss of normal lordosis

11. Amount of cord compression (maximum canal compromise) ____________

12. K Line (Virtual line between the midpoints of the anteroposterior canal diameter at C2 and C7, see example below)

☐ K Line (+)

☐ K Line (-)

Name and signature of person completing the form: Date: D D / M M / Y Y Y Y

mJOA

Patient Details

Patient's Study Number: ☐☐☐ **Date of Birth:** M M / Y Y Y Y

This form was completed on: D D / M M / Y Y Y Y ________________ **visit**

1. Motor dysfunction score of the upper extremity
   - ☐ 0 - Inability to move hands
   - ☐ 1 - Inability to eat with a spoon, but able to move hands
   - ☐ 2 - Inability to button shirt, but able to eat with a spoon
   - ☐ 3 - Able to button shirt with great difficulty
   - ☐ 4 - Able to button shirt with slight difficulty
   - ☐ 5 - No dysfunction

2. Motor dysfunction score of the lower extremity
   - ☐ 0 - Complete loss of motor and sensory function
   - ☐ 1 - Sensory preservation without ability to move legs
   - ☐ 2 - Able to move legs, but unable to walk
   - ☐ 3 - Able to walk on flat floor with a walking aid (cane or crutch)
   - ☐ 4 - Able to walk up and/or down stairs with hand rail
   - ☐ 5 - Moderate-to-significant lack of stability, but able to walk up and/or down stairs w/o hand rail
   - ☐ 6 - Mild lack of stability but walks with smooth reciprocation unaided
   - ☐ 7 - No dysfunction

3. Sensory dysfunction score of the upper extremity
   - ☐ 0 - Complete loss of hand sensation
   - ☐ 1 - Severe sensory loss or pain
   - ☐ 2 - Mild sensory loss
   - ☐ 3 - No sensory loss

4. Sphincter dysfunction score
   - ☐ 0 - Inability to micturate voluntarily
   - ☐ 1 - Marked difficulty with micturition
   - ☐ 2 - Mild to moderate difficulty with micturition
   - ☐ 3 - Normal micturition

mJOA score (0-18) ☐☐

Name and signature of person completing the form:		Date: D D / M M / Y Y Y Y

Neck Disability Index

Patient Details

Patient's Study Number: ☐☐☐ **Date of Birth:** M M / Y Y Y Y

This form was completed on: D D / M M / Y Y Y Y ________________ **visit**

Section 1: Pain Intensity

- ☐ 0 - I have no pain at the moment
- ☐ 1 - The pain is very mild at the moment
- ☐ 2 - The pain is moderate at the moment
- ☐ 3 - The pain is fairly severe at the moment
- ☐ 4 - The pain is very severe at the moment
- ☐ 5 - The pain is the worst imaginable at the moment

Section 2: Personal Care (Washing, dressing, etc.)

- ☐ 0 - I can look after myself normally without causing extra pain
- ☐ 1 - I can look after myself normally but it causes extra pain
- ☐ 2 - It is painful to look after myself and I am slow and careful
- ☐ 3 - I need some help but can manage most of my personal care
- ☐ 4 - I need help every day in most aspects of self care
- ☐ 5 - I do not get dressed, I wash with difficulty and stay in bed

Section 3: Lifting

- ☐ 0 - I can lift heavy weights without extra pain
- ☐ 1 - I can lift heavy weights but it gives extra pain
- ☐ 2 - Pain prevents me lifting heavy weights off the floor, but I can manage if they are conveniently placed, for example on a table
- ☐ 3 - Pain prevents me from lifting heavy weights but I can manage light to medium weights if they are conveniently placed
- ☐ 4 - I can only lift very light weights
- ☐ 5 - I cannot lift or carry anything

Section 4: Reading

- ☐ 0 - I can read as much as I want to with no pain in my neck
- ☐ 1 - I can read as much as I want to with slight pain in my neck
- ☐ 2 - I can read as much as I want to with moderate pain in my neck
- ☐ 3 - I can't read as much as I want to because of moderate pain in my neck
- ☐ 4 - I can hardly read at all because of severe pain in my neck
- ☐ 5 - I cannot read at all

Name and signature of person completing the form:		Date: D D / M M / Y Y Y Y

Neck Disability Index

Patient Details

Patient's Study Number: ☐☐☐ **Date of Birth:** M M / Y Y Y Y

Section 5: Headaches

- ☐ 0 - I have no headaches at all
- ☐ 1 - I have slight headaches which come infrequently
- ☐ 2 - I have moderate headaches which come infrequently
- ☐ 3 - I have moderate headaches which come frequently
- ☐ 4 - I have severe headaches which come frequently
- ☐ 5 - I have headaches almost all the time

Section 6: Concentration

- ☐ 0 - I can concentrate fully when I want to with no difficulty
- ☐ 1 - I can concentrate fully when I want to with slight difficulty
- ☐ 2 - I have a fair degree of difficulty in concentrating when I want to
- ☐ 3 - I have a lot of difficulty in concentrating when I want to
- ☐ 4 - I have a great deal of difficulty in concentrating when I want to
- ☐ 5 - I cannot concentrate at all

Section 7: Work

- ☐ 0 - I can do as much work as I want to
- ☐ 1 - I can only do my usual work, but no more
- ☐ 2 - I can do most of my usual work, but no more
- ☐ 3 - I cannot do my usual work
- ☐ 4 - I can hardly do any work at all
- ☐ 5 - I can't do any work at all

Section 8: Driving

- ☐ 0 - I can drive my car without any neck pain
- ☐ 1 - I can drive my car as long as I want to with slight pain in my neck
- ☐ 2 - I can drive my car as long as I want to with moderate pain in my neck
- ☐ 3 - I can't drive my car as long as I want to because of moderate pain in my neck
- ☐ 4 - I can hardly drive at all because of severe pain in my neck
- ☐ 5 - I can't drive my car at all

Name and signature of person completing the form:		Date: D D / M M / Y Y Y Y

Neck Disability Index

Patient Details

Patient's Study Number: ☐☐☐ **Date of Birth:** M M / Y Y Y Y

Section 9: Sleeping

- ☐ 0 - I have no trouble sleeping
- ☐ 1 - My sleep is slightly disturbed (less than 1 hr sleepless)
- ☐ 2 - My sleep is mildly disturbed (1-2 hrs sleepless)
- ☐ 3 - My sleep is moderately disturbed (2-3 hrs sleepless)
- ☐ 4 - My sleep is greatly disturbed (3-5 hrs sleepless)
- ☐ 5 - My sleep is completely disturbed (5-7 hrs sleepless)

Section 10: Recreation

- ☐ 0 - I am able to engage in all my recreation activities with no neck pain at all
- ☐ 1 - I am able to engage in all my recreation activities, with some pain in my neck
- ☐ 2 - I am able to engage in most, but not all of my usual recreation activities because of pain in my neck
- ☐ 3 - I am able to engage in a few of my usual recreation activities because of pain in my neck
- ☐ 4 - I can hardly do any recreation activities because of pain in my neck
- ☐ 5 - I can't do any recreation activities at all

Name and signature of person completing the form:		Date: D D / M M / Y Y Y Y

Patient Details			
Patient Trial Number	N ☐☐☐	Date of Birth	M M / Y Y Y Y

SF-36v2 Cover page

This form was completed at: ________________ Visit

Visit date: D D / M M / Y Y Y Y

Name and signature of person completing the form		Date	D D / M M / Y Y Y Y

Patient Details			
Patient Trial Number	N ☐☐☐	Date of Birth	M M Y Y Y Y

Your Health and Well-Being

This survey asks for your views about your health. This information will help keep track of how you feel and how well you are able to do your usual activities. ***Thank you for completing this survey!***

For each of the following questions, please tick the one box that best describes your answer.

1. In general, would you say your health is:

Excellent	Very good	Good	Fair	Poor
☐ 1	☐ 2	☐ 3	☐ 4	☐ 5

2. Compared to one year ago, how would you rate your health in general now?

Much better now than one year ago	Somewhat better now than one year ago	About the same as one year ago	Somewhat worse now than one year ago	Much worse now than one year ago
☐ 1	☐ 2	☐ 3	☐ 4	☐ 5

SF-36v2® Health Survey © 1992, 2002, 2009 Medical Outcomes Trust and QualityMetric Incorporated. All rights reserved.
SF-36® is a registered trademark of Medical Outcomes Trust.
(SF-36v2® Health Survey Standard, United Kingdom (English))

Patient Details				
Patient Trial Number	N ☐☐☐	Date of Birth	M M	Y Y Y Y

3. The following questions are about activities you might do during a typical day. Does <u>your health now limit you</u> in these activities? If so, how much?

	Yes, limited a lot	Yes, limited a little	No, not limited at all
a <u>Vigorous activities</u>, such as running, lifting heavy objects, participating in strenuous sports	☐ 1	☐ 2	☐ 3
b <u>Moderate activities</u>, such as moving a table, pushing a vacuum cleaner, bowling, or playing golf	☐ 1	☐ 2	☐ 3
c Lifting or carrying groceries	☐ 1	☐ 2	☐ 3
d Climbing <u>several</u> flights of stairs	☐ 1	☐ 2	☐ 3
e Climbing <u>one</u> flight of stairs	☐ 1	☐ 2	☐ 3
f Bending, kneeling, or stooping	☐ 1	☐ 2	☐ 3
g Walking <u>more than a mile</u>	☐ 1	☐ 2	☐ 3
h Walking <u>several hundred yards</u>	☐ 1	☐ 2	☐ 3
i Walking <u>one hundred yards</u>	☐ 1	☐ 2	☐ 3
j Bathing or dressing yourself	☐ 1	☐ 2	☐ 3

SF-36v2® Health Survey © 1992, 2002, 2009 Medical Outcomes Trust and QualityMetric Incorporated. All rights reserved.
SF-36® is a registered trademark of Medical Outcomes Trust.
(SF-36v2® Health Survey Standard, United Kingdom (English))

Patient Details			
Patient Trial Number	N ☐☐☐	Date of Birth	M M Y Y Y Y

4. During the <u>past 4 weeks</u>, how much of the time have you had any of the following problems with your work or other regular daily activities <u>as a result of your physical health</u>?

	All of the time	Most of the time	Some of the time	A little of the time	None of the time
a Cut down on the <u>amount of time</u> you spent on work or other activities	☐ 1	☐ 2	☐ 3	☐ 4	☐ 5
b <u>Accomplished less</u> than you would like	☐ 1	☐ 2	☐ 3	☐ 4	☐ 5
c Were limited in the <u>kind</u> of work or other activities	☐ 1	☐ 2	☐ 3	☐ 4	☐ 5
d Had <u>difficulty</u> performing the work or other activities (for example, it took extra effort)	☐ 1	☐ 2	☐ 3	☐ 4	☐ 5

5. During the <u>past 4 weeks</u>, how much of the time have you had any of the following problems with your work or other regular daily activities <u>as a result of any emotional problems</u> (such as feeling depressed or anxious)?

	All of the time	Most of the time	Some of the time	A little of the time	None of the time
a Cut down on the <u>amount of time</u> you spent on work or other activities	☐ 1	☐ 2	☐ 3	☐ 4	☐ 5
b <u>Accomplished less</u> than you would like	☐ 1	☐ 2	☐ 3	☐ 4	☐ 5
c Did work or other activities <u>less carefully than usual</u>	☐ 1	☐ 2	☐ 3	☐ 4	☐ 5

SF-36v2® Health Survey © 1992, 2002, 2009 Medical Outcomes Trust and QualityMetric Incorporated. All rights reserved.
SF-36® is a registered trademark of Medical Outcomes Trust.
(SF-36v2® Health Survey Standard, United Kingdom (English))

Patient Details			
Patient Trial Number	N ☐☐☐	Date of Birth	M M Y Y Y Y

6. **During the <u>past 4 weeks</u>, to what extent has your physical health or emotional problems interfered with your normal social activities with family, friends, neighbours, or groups?**

Not at all	Slightly	Moderately	Quite a bit	Extremely
☐ 1	☐ 2	☐ 3	☐ 4	☐ 5

7. **How much <u>bodily</u> pain have you had during the <u>past 4 weeks</u>?**

None	Very mild	Mild	Moderate	Severe	Very severe
☐ 1	☐ 2	☐ 3	☐ 4	☐ 5	☐ 6

8. **During the <u>past 4 weeks</u>, how much did <u>pain</u> interfere with your normal work (including both work outside the home and housework)?**

Not at all	A little bit	Moderately	Quite a bit	Extremely
☐ 1	☐ 2	☐ 3	☐ 4	☐ 5

SF-36v2® Health Survey © 1992, 2002, 2009 Medical Outcomes Trust and QualityMetric Incorporated. All rights reserved.
SF-36® is a registered trademark of Medical Outcomes Trust.
(SF-36v2® Health Survey Standard, United Kingdom (English))

Patient Details				
Patient Trial Number	N ☐☐☐	Date of Birth	M M	Y Y Y Y

9. These questions are about how you feel and how things have been with you <u>during the past 4 weeks</u>. For each question, please give the one answer that comes closest to the way you have been feeling. How much of the time during the <u>past 4 weeks</u>…

	All of the time	Most of the time	Some of the time	A little of the time	None of the time
a Did you feel full of life?	☐ 1	☐ 2	☐ 3	☐ 4	☐ 5
b Have you been very nervous?	☐ 1	☐ 2	☐ 3	☐ 4	☐ 5
c Have you felt so down in the dumps that nothing could cheer you up?	☐ 1	☐ 2	☐ 3	☐ 4	☐ 5
d Have you felt calm and peaceful?	☐ 1	☐ 2	☐ 3	☐ 4	☐ 5
e Did you have a lot of energy?	☐ 1	☐ 2	☐ 3	☐ 4	☐ 5
f Have you felt downhearted and depressed?	☐ 1	☐ 2	☐ 3	☐ 4	☐ 5
g Did you feel worn out?	☐ 1	☐ 2	☐ 3	☐ 4	☐ 5
h Have you been happy?	☐ 1	☐ 2	☐ 3	☐ 4	☐ 5
i Did you feel tired?	☐ 1	☐ 2	☐ 3	☐ 4	☐ 5

10. During the <u>past 4 weeks</u>, how much of the time has your <u>physical health or emotional problems</u> interfered with your social activities (like visiting with friends, relatives, etc.)?

All of the time	Most of the time	Some of the time	A little of the time	None of the time
☐ 1	☐ 2	☐ 3	☐ 4	☐ 5

SF-36v2® Health Survey © 1992, 2002, 2009 Medical Outcomes Trust and QualityMetric Incorporated. All rights reserved.
SF-36® is a registered trademark of Medical Outcomes Trust.
(SF-36v2® Health Survey Standard, United Kingdom (English))

Patient Details				
Patient Trial Number	N	☐☐☐	Date of Birth	M M Y Y Y Y

11. How TRUE or FALSE is <u>each</u> of the following statements for you?

	Definitely true	Mostly true	Don't know	Mostly false	Definitely false
a I seem to get sick a little easier than other people	☐ 1	☐ 2	☐ 3	☐ 4	☐ 5
b I am as healthy as anybody I know	☐ 1	☐ 2	☐ 3	☐ 4	☐ 5
c I expect my health to get worse	☐ 1	☐ 2	☐ 3	☐ 4	☐ 5
d My health is excellent	☐ 1	☐ 2	☐ 3	☐ 4	☐ 5

SF-36v2® Health Survey © 1992, 2002, 2009 Medical Outcomes Trust and QualityMetric Incorporated. All rights reserved.
SF-36® is a registered trademark of Medical Outcomes Trust.
(SF-36v2® Health Survey Standard, United Kingdom (English))

Operation details

Patient Details

Patient's Study Number: ☐☐☐ **Date of Birth:** M M / Y Y Y Y

This form was completed on: D D / M M / Y Y Y Y ________________ **visit**

1. Date of operation: D D / M M / Y Y Y Y
2. Operation title: ☐
3. Approach of operation
   - ☐ Anterior
   - ☐ Posterior
   - ☐ Combined
4. Cervical levels (laminae) treated (e.g. C3-5) ☐
5. Nature of inserted instrumentation (e.g., screws, cages, plates; Product brand name; or *No* if none used):

____

6. Primary surgeon experience ☐ years
7. Operation duration ☐ hours
8. Estimated blood loss ☐ ml
9. Was there any intra-operative complication? ☐ Yes ☐ No

   If Yes, please refer to SAVES v2 questionnaire form

10. Use of either of these

10.1 Intra-operative navigation ☐

10.2 Intra-operative neuromonitoring (neurophysiology) ☐

11. Use of a wound drain ☐ Yes ☐ No
12. Post-operation rehabilitation ☐ Yes ☐ No
13. Post-operation physiotherapy ☐ Yes ☐ No

Name and signature of person completing the form:		Date: D D / M M / Y Y Y Y

mJOA

Patient Details

Patient's Study Number: ☐☐☐ **Date of Birth:** M M / Y Y Y Y

This form was completed on: D D / M M / Y Y Y Y ________________ **visit**

1. Motor dysfunction score of the upper extremity
   - ☐ 0 - Inability to move hands
   - ☐ 1 - Inability to eat with a spoon, but able to move hands
   - ☐ 2 - Inability to button shirt, but able to eat with a spoon
   - ☐ 3 - Able to button shirt with great difficulty
   - ☐ 4 - Able to button shirt with slight difficulty
   - ☐ 5 - No dysfunction

2. Motor dysfunction score of the lower extremity
   - ☐ 0 - Complete loss of motor and sensory function
   - ☐ 1 - Sensory preservation without ability to move legs
   - ☐ 2 - Able to move legs, but unable to walk
   - ☐ 3 - Able to walk on flat floor with a walking aid (cane or crutch)
   - ☐ 4 - Able to walk up and/or down stairs with hand rail
   - ☐ 5 - Moderate-to-significant lack of stability, but able to walk up and/or down stairs w/o hand rail
   - ☐ 6 - Mild lack of stability but walks with smooth reciprocation unaided
   - ☐ 7 - No dysfunction

3. Sensory dysfunction score of the upper extremity
   - ☐ 0 - Complete loss of hand sensation
   - ☐ 1 - Severe sensory loss or pain
   - ☐ 2 - Mild sensory loss
   - ☐ 3 - No sensory loss

4. Sphincter dysfunction score
   - ☐ 0 - Inability to micturate voluntarily
   - ☐ 1 - Marked difficulty with micturition
   - ☐ 2 - Mild to moderate difficulty with micturition
   - ☐ 3 - Normal micturition

mJOA score (0-18) ☐☐

Name and signature of person completing the form:		Date: D D / M M / Y Y Y Y

SAVES v2

Patient Details

Patient's Study Number: ☐☐☐ **Date of Birth:** M M / Y Y Y Y

This form was completed on: D D / M M / Y Y Y Y ________________ **visit**

Please use the following grading system to complete questions in section 1 and 2

1 - Adverse event (AE) does not require treatment & has no AE

2 - AE requires minor invasive (e.g., Foley catheter, nasogastric tube) or simple treatment but has no long-term effect

3 - AE requires invasive (e.g., surgery) or complex treatment (e.g., monitored bed) & is most likely to have a temporary (<6 mos) adverse effect on outcome

4 - AE requires invasive (e.g., surgery) or complex treatment (e.g., monitored bed) & is most likely to have a prolonged (>6 mos) adverse effect on outcome*

5 - Significant neural injury (i.e., 1 or more grade deterioration in ASIA grade) or serious life- or limb-threatening event or any sentinel event†

6 - AE resulting in death

ASIA = American Spinal Injury Association.

** Any AE with a functionally significant (i.e., patient reported) and most likely prolonged (> 6 months) adverse effect on outcome, regardless of required treatment (e.g., nerve root injury that cannot be treated), should be Grade 4.*

† A sentinel event is an unexpected, serious life- or limb-threatening event(s) or any event (e.g., wrong level surgery) that necessitates a formal institutional review process and reporting as defined by your specific institution.

Name and signature of person completing the form:		Date: D D / M M / Y Y Y Y

SAVES v2

Patient Details

**Patient's Study Number:**	☐☐☐	**Date of Birth:**	M M / Y Y Y Y

Section 1: Intraoperative AE categories (Please grade if *Yes*)

	Yes	No	Grade
• Allergic reaction	☐	☐	☐
• Anaesthesia related	☐	☐	☐
• Bone implant interface failure requiring revision	☐	☐	☐
• Cardiac	☐	☐	☐
• Cord injury	☐	☐	☐
• Dural tear	☐	☐	☐
• Hardware malposition requiring revision	☐	☐	☐
• Hypotension (systemic <85 mm Hg for 15 min)	☐	☐	☐
• Massive blood loss (>5 L in 24 hrs or >2 L in 3 hrs)	☐	☐	☐
• Nerve root injury	☐	☐	☐
• Pressure sores	☐	☐	☐
• Vascular injury	☐	☐	☐
• Airway/ventilation	☐	☐	☐
• Visceral injury	☐	☐	☐
• Other (please specify): ____	☐	☐	☐

Name and signature of person completing the form:		Date: D D / M M / Y Y Y Y

SAVES v2

Patient Details

Patient's Study Number: ☐☐☐ **Date of Birth:** M M / Y Y Y Y

Section 2: Postoperative AE categories (please grade if *Yes*)

	Yes	No	Grade
• Cardiac arrest/failure/arrhythmia	☐	☐	☐
• Construct failure with loss of correction	☐	☐	☐
• Construct failure without loss of correction	☐	☐	☐
• CSF leak/meningocele	☐	☐	☐
• Deep vein thrombosis	☐	☐	☐
• Deep wound infection	☐	☐	☐
• Delirium	☐	☐	☐
• Dysphagia	☐	☐	☐
• Dysphonia	☐	☐	☐
• Gastrointestinal bleeding	☐	☐	☐
• Hematoma	☐	☐	☐
• Myocardial infarction	☐	☐	☐
• Neurologic deterioration ≥1 motor grade in ASIA motor scale	☐	☐	☐
• Nonunion	☐	☐	☐
• Pneumonia	☐	☐	☐
• Postop neuropathic pain	☐	☐	☐
• Pressure sores	☐	☐	☐
• Pulmonary embolism	☐	☐	☐
• Superficial wound infection	☐	☐	☐
• Systemic infection	☐	☐	☐
• Urinary tract infection	☐	☐	☐
• Wound dehiscence	☐	☐	☐
• Other (please specify): ________	☐	☐	☐

Estimated effect of AE on Length of Stay:

- ☐ None
- ☐ 1–2 days
- ☐ 3–7 days
- ☐ 8–14 days
- ☐ 15–28 days
- ☐ More than 28 days

Name and signature of person completing the form: ________ Date: D D / M M / Y Y Y Y

Neck Disability Index

Patient Details

Patient's Study Number: ☐☐☐ **Date of Birth:** M M / Y Y Y Y

This form was completed on: D D / M M / Y Y Y Y ________________ **visit**

Section 1: Pain Intensity

- ☐ 0 - I have no pain at the moment
- ☐ 1 - The pain is very mild at the moment
- ☐ 2 - The pain is moderate at the moment
- ☐ 3 - The pain is fairly severe at the moment
- ☐ 4 - The pain is very severe at the moment
- ☐ 5 - The pain is the worst imaginable at the moment

Section 2: Personal Care (Washing, dressing, etc.)

- ☐ 0 - I can look after myself normally without causing extra pain
- ☐ 1 - I can look after myself normally but it causes extra pain
- ☐ 2 - It is painful to look after myself and I am slow and careful
- ☐ 3 - I need some help but can manage most of my personal care
- ☐ 4 - I need help every day in most aspects of self care
- ☐ 5 - I do not get dressed, I wash with difficulty and stay in bed

Section 3: Lifting

- ☐ 0 - I can lift heavy weights without extra pain
- ☐ 1 - I can lift heavy weights but it gives extra pain
- ☐ 2 - Pain prevents me lifting heavy weights off the floor, but I can manage if they are conveniently placed, for example on a table
- ☐ 3 - Pain prevents me from lifting heavy weights but I can manage light to medium weights if they are conveniently placed
- ☐ 4 - I can only lift very light weights
- ☐ 5 - I cannot lift or carry anything

Section 4: Reading

- ☐ 0 - I can read as much as I want to with no pain in my neck
- ☐ 1 - I can read as much as I want to with slight pain in my neck
- ☐ 2 - I can read as much as I want to with moderate pain in my neck
- ☐ 3 - I can't read as much as I want to because of moderate pain in my neck
- ☐ 4 - I can hardly read at all because of severe pain in my neck
- ☐ 5 - I cannot read at all

Name and signature of person completing the form:		Date: D D / M M / Y Y Y Y

Neck Disability Index

Patient Details

Patient's Study Number: ☐☐☐ **Date of Birth:** M M / Y Y Y Y

Section 5: Headaches

- ☐ 0 - I have no headaches at all
- ☐ 1 - I have slight headaches which come infrequently
- ☐ 2 - I have moderate headaches which come infrequently
- ☐ 3 - I have moderate headaches which come frequently
- ☐ 4 - I have severe headaches which come frequently
- ☐ 5 - I have headaches almost all the time

Section 6: Concentration

- ☐ 0 - I can concentrate fully when I want to with no difficulty
- ☐ 1 - I can concentrate fully when I want to with slight difficulty
- ☐ 2 - I have a fair degree of difficulty in concentrating when I want to
- ☐ 3 - I have a lot of difficulty in concentrating when I want to
- ☐ 4 - I have a great deal of difficulty in concentrating when I want to
- ☐ 5 - I cannot concentrate at all

Section 7: Work

- ☐ 0 - I can do as much work as I want to
- ☐ 1 - I can only do my usual work, but no more
- ☐ 2 - I can do most of my usual work, but no more
- ☐ 3 - I cannot do my usual work
- ☐ 4 - I can hardly do any work at all
- ☐ 5 - I can't do any work at all

Section 8: Driving

- ☐ 0 - I can drive my car without any neck pain
- ☐ 1 - I can drive my car as long as I want to with slight pain in my neck
- ☐ 2 - I can drive my car as long as I want to with moderate pain in my neck
- ☐ 3 - I can't drive my car as long as I want to because of moderate pain in my neck
- ☐ 4 - I can hardly drive at all because of severe pain in my neck
- ☐ 5 - I can't drive my car at all

Name and signature of person completing the form: ______ Date: D D / M M / Y Y Y Y

Neck Disability Index

Patient Details

Patient's Study Number: ☐☐☐ **Date of Birth:** M M / Y Y Y Y

Section 9: Sleeping

☐ 0 - I have no trouble sleeping
☐ 1 - My sleep is slightly disturbed (less than 1 hr sleepless)
☐ 2 - My sleep is mildly disturbed (1-2 hrs sleepless)
☐ 3 - My sleep is moderately disturbed (2-3 hrs sleepless)
☐ 4 - My sleep is greatly disturbed (3-5 hrs sleepless)
☐ 5 - My sleep is completely disturbed (5-7 hrs sleepless)

Section 10: Recreation

☐ 0 - I am able to engage in all my recreation activities with no neck pain at all
☐ 1 - I am able to engage in all my recreation activities, with some pain in my neck
☐ 2 - I am able to engage in most, but not all of my usual recreation activities because of pain in my neck
☐ 3 - I am able to engage in a few of my usual recreation activities because of pain in my neck
☐ 4 - I can hardly do any recreation activities because of pain in my neck
☐ 5 - I can't do any recreation activities at all

Name and signature of person completing the form: | Date: D D / M M / Y Y Y Y

Patient Details			
Patient Trial Number	N ☐☐☐	Date of Birth	M M / Y Y Y Y

SF-36v2 Cover page

This form was completed at: ________________ Visit

Visit date: D D / M M / Y Y Y Y

Name and signature of person completing the form		Date D D / M M / Y Y Y Y

Patient Details				
Patient Trial Number	N	☐☐☐	Date of Birth	M M Y Y Y Y

Your Health and Well-Being

This survey asks for your views about your health. This information will help keep track of how you feel and how well you are able to do your usual activities. ***Thank you for completing this survey!***

For each of the following questions, please tick the one box that best describes your answer.

1. In general, would you say your health is:

Excellent	Very good	Good	Fair	Poor
☐ 1	☐ 2	☐ 3	☐ 4	☐ 5

2. <u>Compared to one year ago</u>, how would you rate your health in general <u>now</u>?

Much better now than one year ago	Somewhat better now than one year ago	About the same as one year ago	Somewhat worse now than one year ago	Much worse now than one year ago
☐ 1	☐ 2	☐ 3	☐ 4	☐ 5

SF-36v2® Health Survey © 1992, 2002, 2009 Medical Outcomes Trust and QualityMetric Incorporated. All rights reserved.
SF-36® is a registered trademark of Medical Outcomes Trust.
(SF-36v2® Health Survey Standard, United Kingdom (English))

Patient Details			
Patient Trial Number	N	Date of Birth	MM YYYY

3. **The following questions are about activities you might do during a typical day. Does <u>your health now limit you</u> in these activities? If so, how much?**

	Yes, limited a lot	Yes, limited a little	No, not limited at all
a <u>Vigorous activities</u>, such as running, lifting heavy objects, participating in strenuous sports	☐ 1	☐ 2	☐ 3
b <u>Moderate activities</u>, such as moving a table, pushing a vacuum cleaner, bowling, or playing golf	☐ 1	☐ 2	☐ 3
c Lifting or carrying groceries	☐ 1	☐ 2	☐ 3
d Climbing <u>several</u> flights of stairs	☐ 1	☐ 2	☐ 3
e Climbing <u>one</u> flight of stairs	☐ 1	☐ 2	☐ 3
f Bending, kneeling, or stooping	☐ 1	☐ 2	☐ 3
g Walking <u>more than a mile</u>	☐ 1	☐ 2	☐ 3
h Walking <u>several hundred yards</u>	☐ 1	☐ 2	☐ 3
i Walking <u>one hundred yards</u>	☐ 1	☐ 2	☐ 3
j Bathing or dressing yourself	☐ 1	☐ 2	☐ 3

SF-36v2® Health Survey © 1992, 2002, 2009 Medical Outcomes Trust and QualityMetric Incorporated. All rights reserved.
SF-36® is a registered trademark of Medical Outcomes Trust.
(SF-36v2® Health Survey Standard, United Kingdom (English))

Patient Details				
Patient Trial Number	N	☐☐☐	Date of Birth	M M / Y Y Y Y

4. During the <u>past 4 weeks</u>, how much of the time have you had any of the following problems with your work or other regular daily activities <u>as a result of your physical health</u>?

	All of the time	Most of the time	Some of the time	A little of the time	None of the time
a Cut down on the amount of time you spent on work or other activities	☐ 1	☐ 2	☐ 3	☐ 4	☐ 5
b Accomplished less than you would like	☐ 1	☐ 2	☐ 3	☐ 4	☐ 5
c Were limited in the kind of work or other activities	☐ 1	☐ 2	☐ 3	☐ 4	☐ 5
d Had difficulty performing the work or other activities (for example, it took extra effort)	☐ 1	☐ 2	☐ 3	☐ 4	☐ 5

5. During the <u>past 4 weeks</u>, how much of the time have you had any of the following problems with your work or other regular daily activities <u>as a result of any emotional problems</u> (such as feeling depressed or anxious)?

	All of the time	Most of the time	Some of the time	A little of the time	None of the time
a Cut down on the amount of time you spent on work or other activities	☐ 1	☐ 2	☐ 3	☐ 4	☐ 5
b Accomplished less than you would like	☐ 1	☐ 2	☐ 3	☐ 4	☐ 5
c Did work or other activities less carefully than usual	☐ 1	☐ 2	☐ 3	☐ 4	☐ 5

SF-36v2® Health Survey © 1992, 2002, 2009 Medical Outcomes Trust and QualityMetric Incorporated. All rights reserved.
SF-36® is a registered trademark of Medical Outcomes Trust.
(SF-36v2® Health Survey Standard, United Kingdom (English))

Patient Details			
Patient Trial Number	N	Date of Birth	M M Y Y Y Y

6. **During the <u>past 4 weeks</u>, to what extent has your physical health or emotional problems interfered with your normal social activities with family, friends, neighbours, or groups?**

Not at all	Slightly	Moderately	Quite a bit	Extremely
☐ 1	☐ 2	☐ 3	☐ 4	☐ 5

7. **How much <u>bodily</u> pain have you had during the <u>past 4 weeks</u>?**

None	Very mild	Mild	Moderate	Severe	Very severe
☐ 1	☐ 2	☐ 3	☐ 4	☐ 5	☐ 6

8. **During the <u>past 4 weeks</u>, how much did <u>pain</u> interfere with your normal work (including both work outside the home and housework)?**

Not at all	A little bit	Moderately	Quite a bit	Extremely
☐ 1	☐ 2	☐ 3	☐ 4	☐ 5

SF-36v2® Health Survey © 1992, 2002, 2009 Medical Outcomes Trust and QualityMetric Incorporated. All rights reserved.
SF-36® is a registered trademark of Medical Outcomes Trust.
(SF-36v2® Health Survey Standard, United Kingdom (English))

Patient Details			
Patient Trial Number	N	Date of Birth	MM YYYY

9. **These questions are about how you feel and how things have been with you <u>during the past 4 weeks</u>. For each question, please give the one answer that comes closest to the way you have been feeling. How much of the time during the <u>past 4 weeks</u>…**

	All of the time	Most of the time	Some of the time	A little of the time	None of the time
a Did you feel full of life?	☐ 1	☐ 2	☐ 3	☐ 4	☐ 5
b Have you been very nervous?	☐ 1	☐ 2	☐ 3	☐ 4	☐ 5
c Have you felt so down in the dumps that nothing could cheer you up?	☐ 1	☐ 2	☐ 3	☐ 4	☐ 5
d Have you felt calm and peaceful?	☐ 1	☐ 2	☐ 3	☐ 4	☐ 5
e Did you have a lot of energy?	☐ 1	☐ 2	☐ 3	☐ 4	☐ 5
f Have you felt downhearted and depressed?	☐ 1	☐ 2	☐ 3	☐ 4	☐ 5
g Did you feel worn out?	☐ 1	☐ 2	☐ 3	☐ 4	☐ 5
h Have you been happy?	☐ 1	☐ 2	☐ 3	☐ 4	☐ 5
i Did you feel tired?	☐ 1	☐ 2	☐ 3	☐ 4	☐ 5

10. **During the <u>past 4 weeks</u>, how much of the time has your <u>physical health or emotional problems</u> interfered with your social activities (like visiting with friends, relatives, etc.)?**

All of the time	Most of the time	Some of the time	A little of the time	None of the time
☐ 1	☐ 2	☐ 3	☐ 4	☐ 5

SF-36v2® Health Survey © 1992, 2002, 2009 Medical Outcomes Trust and QualityMetric Incorporated. All rights reserved.
SF-36® is a registered trademark of Medical Outcomes Trust.
(SF-36v2® Health Survey Standard, United Kingdom (English))

Patient Details			
Patient Trial Number	N ☐☐☐	Date of Birth	M M Y Y Y Y

11. How TRUE or FALSE is <u>each</u> of the following statements for you?

	Definitely true	Mostly true	Don't know	Mostly false	Definitely false
a I seem to get sick a little easier than other people	☐ 1	☐ 2	☐ 3	☐ 4	☐ 5
b I am as healthy as anybody I know	☐ 1	☐ 2	☐ 3	☐ 4	☐ 5
c I expect my health to get worse	☐ 1	☐ 2	☐ 3	☐ 4	☐ 5
d My health is excellent	☐ 1	☐ 2	☐ 3	☐ 4	☐ 5

SF-36v2® Health Survey © 1992, 2002, 2009 Medical Outcomes Trust and QualityMetric Incorporated. All rights reserved.
SF-36® is a registered trademark of Medical Outcomes Trust.
(SF-36v2® Health Survey Standard, United Kingdom (English))

mJOA

Patient Details

Patient's Study Number: ☐☐☐ **Date of Birth:** M M / Y Y Y Y

This form was completed on: D D / M M / Y Y Y Y ________________ **visit**

1. Motor dysfunction score of the upper extremity
   - ☐ 0 - Inability to move hands
   - ☐ 1 - Inability to eat with a spoon, but able to move hands
   - ☐ 2 - Inability to button shirt, but able to eat with a spoon
   - ☐ 3 - Able to button shirt with great difficulty
   - ☐ 4 - Able to button shirt with slight difficulty
   - ☐ 5 - No dysfunction

2. Motor dysfunction score of the lower extremity
   - ☐ 0 - Complete loss of motor and sensory function
   - ☐ 1 - Sensory preservation without ability to move legs
   - ☐ 2 - Able to move legs, but unable to walk
   - ☐ 3 - Able to walk on flat floor with a walking aid (cane or crutch)
   - ☐ 4 - Able to walk up and/or down stairs with hand rail
   - ☐ 5 - Moderate-to-significant lack of stability, but able to walk up and/or down stairs w/o hand rail
   - ☐ 6 - Mild lack of stability but walks with smooth reciprocation unaided
   - ☐ 7 - No dysfunction

3. Sensory dysfunction score of the upper extremity
   - ☐ 0 - Complete loss of hand sensation
   - ☐ 1 - Severe sensory loss or pain
   - ☐ 2 - Mild sensory loss
   - ☐ 3 - No sensory loss

4. Sphincter dysfunction score
   - ☐ 0 - Inability to micturate voluntarily
   - ☐ 1 - Marked difficulty with micturition
   - ☐ 2 - Mild to moderate difficulty with micturition
   - ☐ 3 - Normal micturition

mJOA score (0-18) ☐☐

Name and signature of person completing the form:		Date: D D / M M / Y Y Y Y

SAVES v2

Patient Details

Patient's Study Number: ☐☐☐ **Date of Birth:** M M / Y Y Y Y

This form was completed on: D D / M M / Y Y Y Y ________________ **visit**

Please use the following grading system to complete questions in next page

1 - Adverse event (AE) does not require treatment & has no AE

2 - AE requires minor invasive (e.g., Foley catheter, nasogastric tube) or simple treatment but has no long-term effect

3 - AE requires invasive (e.g., surgery) or complex treatment (e.g., monitored bed) & is most likely to have a temporary (<6 mos) adverse effect on outcome

4 - AE requires invasive (e.g., surgery) or complex treatment (e.g., monitored bed) & is most likely to have a prolonged (>6 mos) adverse effect on outcome*

5 - Significant neural injury (i.e., 1 or more grade deterioration in ASIA grade) or serious life- or limb-threatening event or any sentinel event†

6 - AE resulting in death

ASIA = American Spinal Injury Association.

** Any AE with a functionally significant (i.e., patient reported) and most likely prolonged (> 6 months) adverse effect on outcome, regardless of required treatment (e.g., nerve root injury that cannot be treated), should be Grade 4.*

† A sentinel event is an unexpected, serious life- or limb-threatening event(s) or any event (e.g., wrong level surgery) that necessitates a formal institutional review process and reporting as defined by your specific institution.

Name and signature of person completing the form:		Date: D D / M M / Y Y Y Y

SAVES v2

Patient Details

Patient's Study Number: ☐☐☐ **Date of Birth:** M M / Y Y Y Y

Postoperative AE categories (please grade if *Yes*)

	Yes	No	Grade
• Cardiac arrest/failure/arrhythmia	☐	☐	☐
• Construct failure with loss of correction	☐	☐	☐
• Construct failure without loss of correction	☐	☐	☐
• CSF leak/meningocele	☐	☐	☐
• Deep vein thrombosis	☐	☐	☐
• Deep wound infection	☐	☐	☐
• Delirium	☐	☐	☐
• Dysphagia	☐	☐	☐
• Dysphonia	☐	☐	☐
• Gastrointestinal bleeding	☐	☐	☐
• Hematoma	☐	☐	☐
• Myocardial infarction	☐	☐	☐
• Neurologic deterioration ≥1 motor grade in ASIA motor scale	☐	☐	☐
• Nonunion	☐	☐	☐
• Pneumonia	☐	☐	☐
• Postop neuropathic pain	☐	☐	☐
• Pressure sores	☐	☐	☐
• Pulmonary embolism	☐	☐	☐
• Superficial wound infection	☐	☐	☐
• Systemic infection	☐	☐	☐
• Urinary tract infection	☐	☐	☐
• Wound dehiscence	☐	☐	☐
• Other (please specify): ______	☐	☐	☐

Estimated effect of AE on Length of Stay:

☐ None
☐ 1–2 days
☐ 3–7 days
☐ 8–14 days
☐ 15–28 days
☐ More than 28 days

Name and signature of person completing the form: ______ Date: D D / M M / Y Y Y Y

Neck Disability Index

Patient Details

Patient's Study Number: ☐☐☐ **Date of Birth:** M M / Y Y Y Y

This form was completed on: D D / M M / Y Y Y Y ________________ **visit**

Section 1: Pain Intensity

- ☐ 0 - I have no pain at the moment
- ☐ 1 - The pain is very mild at the moment
- ☐ 2 - The pain is moderate at the moment
- ☐ 3 - The pain is fairly severe at the moment
- ☐ 4 - The pain is very severe at the moment
- ☐ 5 - The pain is the worst imaginable at the moment

Section 2: Personal Care (Washing, dressing, etc.)

- ☐ 0 - I can look after myself normally without causing extra pain
- ☐ 1 - I can look after myself normally but it causes extra pain
- ☐ 2 - It is painful to look after myself and I am slow and careful
- ☐ 3 - I need some help but can manage most of my personal care
- ☐ 4 - I need help every day in most aspects of self care
- ☐ 5 - I do not get dressed, I wash with difficulty and stay in bed

Section 3: Lifting

- ☐ 0 - I can lift heavy weights without extra pain
- ☐ 1 - I can lift heavy weights but it gives extra pain
- ☐ 2 - Pain prevents me lifting heavy weights off the floor, but I can manage if they are conveniently placed, for example on a table
- ☐ 3 - Pain prevents me from lifting heavy weights but I can manage light to medium weights if they are conveniently placed
- ☐ 4 - I can only lift very light weights
- ☐ 5 - I cannot lift or carry anything

Section 4: Reading

- ☐ 0 - I can read as much as I want to with no pain in my neck
- ☐ 1 - I can read as much as I want to with slight pain in my neck
- ☐ 2 - I can read as much as I want to with moderate pain in my neck
- ☐ 3 - I can't read as much as I want to because of moderate pain in my neck
- ☐ 4 - I can hardly read at all because of severe pain in my neck
- ☐ 5 - I cannot read at all

Name and signature of person completing the form:		Date: D D / M M / Y Y Y Y

Neck Disability Index

Patient Details

Patient's Study Number: ☐☐☐ **Date of Birth:** M M / Y Y Y Y

Section 5: Headaches

- ☐ 0 - I have no headaches at all
- ☐ 1 - I have slight headaches which come infrequently
- ☐ 2 - I have moderate headaches which come infrequently
- ☐ 3 - I have moderate headaches which come frequently
- ☐ 4 - I have severe headaches which come frequently
- ☐ 5 - I have headaches almost all the time

Section 6: Concentration

- ☐ 0 - I can concentrate fully when I want to with no difficulty
- ☐ 1 - I can concentrate fully when I want to with slight difficulty
- ☐ 2 - I have a fair degree of difficulty in concentrating when I want to
- ☐ 3 - I have a lot of difficulty in concentrating when I want to
- ☐ 4 - I have a great deal of difficulty in concentrating when I want to
- ☐ 5 - I cannot concentrate at all

Section 7: Work

- ☐ 0 - I can do as much work as I want to
- ☐ 1 - I can only do my usual work, but no more
- ☐ 2 - I can do most of my usual work, but no more
- ☐ 3 - I cannot do my usual work
- ☐ 4 - I can hardly do any work at all
- ☐ 5 - I can't do any work at all

Section 8: Driving

- ☐ 0 - I can drive my car without any neck pain
- ☐ 1 - I can drive my car as long as I want to with slight pain in my neck
- ☐ 2 - I can drive my car as long as I want to with moderate pain in my neck
- ☐ 3 - I can't drive my car as long as I want to because of moderate pain in my neck
- ☐ 4 - I can hardly drive at all because of severe pain in my neck
- ☐ 5 - I can't drive my car at all

Name and signature of person completing the form:		Date: D D / M M / Y Y Y Y

Neck Disability Index

Patient Details

Patient's Study Number: ☐☐☐ **Date of Birth:** M M / Y Y Y Y

Section 9: Sleeping

- ☐ 0 - I have no trouble sleeping
- ☐ 1 - My sleep is slightly disturbed (less than 1 hr sleepless)
- ☐ 2 - My sleep is mildly disturbed (1-2 hrs sleepless)
- ☐ 3 - My sleep is moderately disturbed (2-3 hrs sleepless)
- ☐ 4 - My sleep is greatly disturbed (3-5 hrs sleepless)
- ☐ 5 - My sleep is completely disturbed (5-7 hrs sleepless)

Section 10: Recreation

- ☐ 0 - I am able to engage in all my recreation activities with no neck pain at all
- ☐ 1 - I am able to engage in all my recreation activities, with some pain in my neck
- ☐ 2 - I am able to engage in most, but not all of my usual recreation activities because of pain in my neck
- ☐ 3 - I am able to engage in a few of my usual recreation activities because of pain in my neck
- ☐ 4 - I can hardly do any recreation activities because of pain in my neck
- ☐ 5 - I can't do any recreation activities at all

Name and signature of person completing the form:		Date: D D / M M / Y Y Y Y

Patient Details			
Patient Trial Number	N ☐☐☐	Date of Birth	M M / Y Y Y Y

SF-36v2 Cover page

This form was completed at: ________________ Visit

Visit date: D D / M M / Y Y Y Y

Name and signature of person completing the form		Date	D D / M M / Y Y Y Y

Patient Details				
Patient Trial Number	N ☐☐☐	Date of Birth	M M	Y Y Y Y

Your Health and Well-Being

This survey asks for your views about your health. This information will help keep track of how you feel and how well you are able to do your usual activities. ***Thank you for completing this survey!***

For each of the following questions, please tick the one box that best describes your answer.

1. In general, would you say your health is:

Excellent	Very good	Good	Fair	Poor
☐ 1	☐ 2	☐ 3	☐ 4	☐ 5

2. <u>Compared to one year ago</u>, how would you rate your health in general <u>now</u>?

Much better now than one year ago	Somewhat better now than one year ago	About the same as one year ago	Somewhat worse now than one year ago	Much worse now than one year ago
☐ 1	☐ 2	☐ 3	☐ 4	☐ 5

SF-36v2® Health Survey © 1992, 2002, 2009 Medical Outcomes Trust and QualityMetric Incorporated. All rights reserved.
SF-36® is a registered trademark of Medical Outcomes Trust.
(SF-36v2® Health Survey Standard, United Kingdom (English))

Patient Details			
Patient Trial Number	N ☐☐☐	Date of Birth	M M Y Y Y Y

3. The following questions are about activities you might do during a typical day. Does <u>your health now limit you</u> in these activities? If so, how much?

	Yes, limited a lot	Yes, limited a little	No, not limited at all
a <u>Vigorous activities</u>, such as running, lifting heavy objects, participating in strenuous sports	☐ 1	☐ 2	☐ 3
b <u>Moderate activities</u>, such as moving a table, pushing a vacuum cleaner, bowling, or playing golf	☐ 1	☐ 2	☐ 3
c Lifting or carrying groceries	☐ 1	☐ 2	☐ 3
d Climbing <u>several</u> flights of stairs	☐ 1	☐ 2	☐ 3
e Climbing <u>one</u> flight of stairs	☐ 1	☐ 2	☐ 3
f Bending, kneeling, or stooping	☐ 1	☐ 2	☐ 3
g Walking <u>more than a mile</u>	☐ 1	☐ 2	☐ 3
h Walking <u>several hundred yards</u>	☐ 1	☐ 2	☐ 3
i Walking <u>one hundred yards</u>	☐ 1	☐ 2	☐ 3
j Bathing or dressing yourself	☐ 1	☐ 2	☐ 3

SF-36v2® Health Survey © 1992, 2002, 2009 Medical Outcomes Trust and QualityMetric Incorporated. All rights reserved.
SF-36® is a registered trademark of Medical Outcomes Trust.
(SF-36v2® Health Survey Standard, United Kingdom (English))

Patient Details				
Patient Trial Number	N ☐☐☐	Date of Birth	M M	Y Y Y Y

4. During the <u>past 4 weeks</u>, how much of the time have you had any of the following problems with your work or other regular daily activities <u>as a result of your physical health</u>?

	All of the time	Most of the time	Some of the time	A little of the time	None of the time
a Cut down on the <u>amount of time</u> you spent on work or other activities	☐ 1	☐ 2	☐ 3	☐ 4	☐ 5
b <u>Accomplished less</u> than you would like	☐ 1	☐ 2	☐ 3	☐ 4	☐ 5
c Were limited in the <u>kind</u> of work or other activities	☐ 1	☐ 2	☐ 3	☐ 4	☐ 5
d Had <u>difficulty</u> performing the work or other activities (for example, it took extra effort)	☐ 1	☐ 2	☐ 3	☐ 4	☐ 5

5. During the <u>past 4 weeks</u>, how much of the time have you had any of the following problems with your work or other regular daily activities <u>as a result of any emotional problems</u> (such as feeling depressed or anxious)?

	All of the time	Most of the time	Some of the time	A little of the time	None of the time
a Cut down on the <u>amount of time</u> you spent on work or other activities	☐ 1	☐ 2	☐ 3	☐ 4	☐ 5
b <u>Accomplished less</u> than you would like	☐ 1	☐ 2	☐ 3	☐ 4	☐ 5
c Did work or other activities <u>less carefully than usual</u>	☐ 1	☐ 2	☐ 3	☐ 4	☐ 5

SF-36v2® Health Survey © 1992, 2002, 2009 Medical Outcomes Trust and QualityMetric Incorporated. All rights reserved.
SF-36® is a registered trademark of Medical Outcomes Trust.
(SF-36v2® Health Survey Standard, United Kingdom (English))

Patient Details				
Patient Trial Number	N ☐☐☐	Date of Birth	MM	YYYY

6. **During the <u>past 4 weeks</u>, to what extent has your physical health or emotional problems interfered with your normal social activities with family, friends, neighbours, or groups?**

Not at all	Slightly	Moderately	Quite a bit	Extremely
☐ 1	☐ 2	☐ 3	☐ 4	☐ 5

7. **How much <u>bodily</u> pain have you had during the <u>past 4 weeks</u>?**

None	Very mild	Mild	Moderate	Severe	Very severe
☐ 1	☐ 2	☐ 3	☐ 4	☐ 5	☐ 6

8. **During the <u>past 4 weeks</u>, how much did <u>pain</u> interfere with your normal work (including both work outside the home and housework)?**

Not at all	A little bit	Moderately	Quite a bit	Extremely
☐ 1	☐ 2	☐ 3	☐ 4	☐ 5

SF-36v2® Health Survey © 1992, 2002, 2009 Medical Outcomes Trust and QualityMetric Incorporated. All rights reserved.
SF-36® is a registered trademark of Medical Outcomes Trust.
(SF-36v2® Health Survey Standard, United Kingdom (English))

Patient Details			
Patient Trial Number	N ☐☐☐	Date of Birth	MM YYYY

9. These questions are about how you feel and how things have been with you <u>during the past 4 weeks</u>. For each question, please give the one answer that comes closest to the way you have been feeling. How much of the time during the <u>past 4 weeks</u>…

	All of the time	Most of the time	Some of the time	A little of the time	None of the time
a Did you feel full of life?	☐ 1	☐ 2	☐ 3	☐ 4	☐ 5
b Have you been very nervous?	☐ 1	☐ 2	☐ 3	☐ 4	☐ 5
c Have you felt so down in the dumps that nothing could cheer you up?	☐ 1	☐ 2	☐ 3	☐ 4	☐ 5
d Have you felt calm and peaceful?	☐ 1	☐ 2	☐ 3	☐ 4	☐ 5
e Did you have a lot of energy?	☐ 1	☐ 2	☐ 3	☐ 4	☐ 5
f Have you felt downhearted and depressed?	☐ 1	☐ 2	☐ 3	☐ 4	☐ 5
g Did you feel worn out?	☐ 1	☐ 2	☐ 3	☐ 4	☐ 5
h Have you been happy?	☐ 1	☐ 2	☐ 3	☐ 4	☐ 5
i Did you feel tired?	☐ 1	☐ 2	☐ 3	☐ 4	☐ 5

10. During the <u>past 4 weeks</u>, how much of the time has your <u>physical health or emotional problems</u> interfered with your social activities (like visiting with friends, relatives, etc.)?

All of the time	Most of the time	Some of the time	A little of the time	None of the time
☐ 1	☐ 2	☐ 3	☐ 4	☐ 5

SF-36v2® Health Survey © 1992, 2002, 2009 Medical Outcomes Trust and QualityMetric Incorporated. All rights reserved.
SF-36® is a registered trademark of Medical Outcomes Trust.
(SF-36v2® Health Survey Standard, United Kingdom (English))

Patient Details			
Patient Trial Number	N ☐☐☐	Date of Birth	M M Y Y Y Y

11. How TRUE or FALSE is each of the following statements for you?

	Definitely true	Mostly true	Don't know	Mostly false	Definitely false
a I seem to get sick a little easier than other people	☐ 1	☐ 2	☐ 3	☐ 4	☐ 5
b I am as healthy as anybody I know	☐ 1	☐ 2	☐ 3	☐ 4	☐ 5
c I expect my health to get worse	☐ 1	☐ 2	☐ 3	☐ 4	☐ 5
d My health is excellent	☐ 1	☐ 2	☐ 3	☐ 4	☐ 5

SF-36v2® Health Survey © 1992, 2002, 2009 Medical Outcomes Trust and QualityMetric Incorporated. All rights reserved.
SF-36® is a registered trademark of Medical Outcomes Trust.
(SF-36v2® Health Survey Standard, United Kingdom (English))
